# Identification and substrate prediction of new *Fragaria x ananassa* aquaporins and expression in different tissues and during strawberry fruit development

**DOI:** 10.1038/s41438-018-0019-0

**Published:** 2018-04-01

**Authors:** Britt Merlaen, Ellen De Keyser, Marie-Christine Van Labeke

**Affiliations:** 1 Plant Production, Faculty of Bioscience Engineering, Ghent University, Coupure Links 653, 9000 Gent, Belgium; 2 Flanders Research Institute for Agriculture Fisheries and Food (ILVO), Plant Sciences Unit, Caritasstraat 39, 9090 Melle, Belgium

## Abstract

The newly identified aquaporin coding sequences presented here pave the way for further insights into the plant–water relations in the commercial strawberry (*Fragaria x ananassa*). Aquaporins are water channel proteins that allow water to cross (intra)cellular membranes. In *Fragaria x ananassa*, few of them have been identified hitherto, hampering the exploration of the water transport regulation at cellular level. Here, we present new aquaporin coding sequences belonging to different subclasses: plasma membrane intrinsic proteins subtype 1 and subtype 2 (PIP1 and PIP2) and tonoplast intrinsic proteins (TIP). The classification is based on phylogenetic analysis and is confirmed by the presence of conserved residues. Substrate-specific signature sequences (SSSSs) and specificity-determining positions (SDPs) predict the substrate specificity of each new aquaporin. Expression profiling in leaves, petioles and developing fruits reveals distinct patterns, even within the same (sub)class. Expression profiles range from leaf-specific expression over constitutive expression to fruit-specific expression. Both upregulation and downregulation during fruit ripening occur. Substrate specificity and expression profiles suggest that functional specialization exists among aquaporins belonging to a different but also to the same (sub)class.

## Strawberry production: Examining cellular water use

Insights into the mechanisms behind cellular water transport within strawberry plants could help optimize crop management and yields. Strawberry plants have shallow root systems, large leaves and produce fruit with a high water content, and so good water management is essential for optimizing commercial strawberry crops. However, the precise mechanisms inherent in cellular water management in strawberries are not clear. Marie-Christine Van Labeke at Ghent University in Belgium, and co-workers identified and coded three sub-classes of cellular water channels called aquaporins in the commercial strawberry, Fragaria x ananassa. Aquaporins form gated pores in cellular membranes, which open and close to control water transport within the plant. The researchers found that aquaporin sub-classes are expressed differently in stalks, leaves and fruits dependent on ripening stage, and, even within the same sub-class, they have individual specialized functions.

## Introduction

Because of its shallow root system, large leaf area and high fruit-water content, good water management is key to strawberry production^1,2^. At the cellular level, water transport is controlled through water channels called aquaporins. Aquaporins form a pore in (intra)cellular membranes and in this way facilitate water transport across these membranes. A growing body of evidence is presenting them as influencing factors when it comes to plant–water relations^3,4^.

Five aquaporin classes are distinguished in higher plants, based on sequence and subcellular localization, although occurrence at different locations has been reported^5–9^. Due to their abundance and subcellular localization and the fact that they generally transport water more efficiently than other types, the plasma membrane intrinsic proteins (PIPs) and the tonoplast intrinsic proteins (TIPs) are most promising when looking for aquaporins that significantly influence the plant–water status^3^. Other classes are nodulin-26 like intrinsic proteins (NIPs), small basic intrinsic proteins (SIPs) and X intrinsic proteins (XIPs), a small, recently discovered class^7,10,11^.

Aquaporins have six transmembrane helices and five loops connecting them. Their 3D structure has a pore in the middle. They form heterotetramers, resulting in units with four pores^12–15^. Next to this conserved 3D structure, aquaporins have several highly conserved residues defining the pore specificity. In the first place, the two conserved NPA motifs (Asparagine–Proline–Alanine) that are in close proximity of each other in the 3D protein configuration aid in directing the water molecules one by one through the channel^16^. Additionally, the Ar/R (an aromatic amino acid and Arginine) selectivity filter also contributes to the specificity of the channel by providing a size barrier and effectuating proton exclusion (reviewed in refs. ^3,8,17–19^). Aquaporins can quickly and reversibly transition from an open to a closed state. This process, called gating, is controlled through phosphorylation and protonation of specific, also highly conserved residues^20–24^.

Aquaporins have been shown to transport several small solutes in addition to water. These include ammonia, arsenite, silicic acid, boron, antimonite, hydrogen peroxide and carbon dioxide^3,8,25^. Arsenite, silicic acid and antimonite are generally transported by aquaporins other than PIPs or TIPs^8,26^. The residues surrounding the NPA motifs and the Ar/R residues, along with the Froger’s P1–P5 residues are involved in discriminating between different substrates^27^. Substrate-specific signature sequences (SSSSs) for these positions have been suggested for different substrates, along with additional specificity-determining positions (SDPs)^8,26^.

Qualitative and quantitative knowledge about the contribution of aquaporins to maintaining the plant–water status is very limited in *Fragaria x ananassa*. In the diploid *Fragaria vesca*, Surbanovski et al. have identified 10 aquaporins, belonging to PIP1, PIP2 and TIP (sub)classes^28^. Only four aquaporins have been identified in the octoploid *F. x ananassa* up to now: a root-specific TIP (FaRB7 (Genbank Acc. No. DQ178022.1))^29^, a PIP subtype 1 (FaPIP1;1 (Genbank Acc. No. GQ390798.1))^30^, a PIP subtype 2 (FaPIP2;1 (Genbank Acc. No. GQ390799.1))^31^ and one NIP (FaNIP1;1 (Genbank Acc. No. KJ159565.1))^32^. Considering the multitude of physiological and biological processes that are affected by cellular water transport and plant–water relations in general, it is in the interest of many research fields related to strawberry cultivation that new *F. x ananassa* aquaporin coding sequences are identified.

In this study, we present several new *F. x ananassa* PIP-coding sequences. As a basis for this, known coding sequences from the wild strawberry (*F. vesca*) were used because of the high homology that exists among aquaporins^28^. Additionally, the recent sequencing project of the octoploid strawberry is a source of new PIP and TIP aquaporin coding sequences^33^. The substrate specificity of the newly identified sequences is predicted based on SSSSs and SDPs. We also analyse the expression of different groups of aquaporins, both PIPs and TIPs, across different tissues and fruit developmental stages. The variety in predicted substrates and expression patterns points at functional specialization, even within (sub)groups. Providing these PIP and TIP coding sequences, this study paves the way for further research on plant–water balance in the commercial strawberry, including research in ripening, abiotic stress and water use.

## Materials and methods

### RNA extraction and reverse transcription

RNA was extracted using a method modified from Chang et al.^34^. Modifications were kindly provided by Kevin Folta (Horticultural Sciences Department, University of Florida). For detailed protocols, please refer to Supplementary file 1.

### Isolation of new PIP-coding sequences

Primers were designed using Primer3 software (http://biotools.umassmed.edu/bioapps/primer3_www.cgi) based on *F. vesca* PIP sequences and a partial *F. x ananassa* coding sequence (Genbank Acc. No. DQ022749.1) (Table S3)^28,35^. These primers were used in PCR on *F. x ananassa* cv. Elsanta cDNA in order to amplify and sequence *F. x ananassa* aquaporin fragments. For detailed protocols, please refer to Supplementary file 1.

In addition to these fragments, an EST (Genbank Acc. No. GW403182.1) was derived from the NCBI database by using the BLAST tool (http://blast.ncbi.nlm.nih.gov/Blast.cgi). The FaPIP1;1 (Genbank Acc. No. GQ390798.1) coding sequence was used as a query to search the expressed sequence tags database of the organism *F. x ananassa*^30^.

Next, 5’ RACE (Rapid amplification of cDNA ends) PCR and 3’ RACE PCR were applied to these fragments and the EST derived from the NCBI database. For detailed protocols, please refer to Supplementary file 1.

Finally, primers in the 5’ and 3’ untranslated region were designed for amplification and sequencing of the full length coding sequence. For detailed protocols, please refer to Supplementary file 1. The resulting sequences were named according to the current plant aquaporin nomenclature^36^.

### PIP and TIP coding sequences in Strawberry GARDEN

*Fragaria vesca* PIP protein sequences (FvPIP1;3, FvPIP2;2, FvPIP2;3, FvPIP2;4, FvPIP2;5, FvPIP2;6 and FvPIP2;7) and predicted TIP protein sequences derived from the NCBI database were used as query sequences to search the FAN/_r1.1_pep database of the Strawberry Genome And Resource Database ENtry (Strawberry GARDEN project) (http://strawberry-garden.kazusa.or.jp/blast.html) by means of the BLASTp tool^28,33^. Coding sequences resulting from this BLAST have names starting with FAN.

### Sequence analysis

Translation of coding sequences into protein sequences was done by means of the online translate tool of the Swiss Institute of Bioinformatics (http://web.expasy.org/translate/). All alignments were performed using either the ClustalX2.1 or CLC program. Phylogenetic trees were constructed by means of Neighbour Joining using the CLC program. The number of transmembrane helices was predicted using the TMHMM software of the Technical University of Denmark (http://www.cbs.dtu.dk/services/TMHMM/). SSSSs of NPA motifs, Ar/R filters (H2, H5, Loop E(1), Loop E(2)), Froger’s positions (P1–P5) and SDPs were identified based on careful visual inspection of multiple sequence alignments of *F. x ananassa* aquaporins and alignments reported earlier^8,27^. Indication of transmembrane helices and conserved residues onto alignments was done using Jalview.

### Plant material for RT-qPCR

Vegetative tissues (young fully developed leaves (Ly), dark green fully developed mature leaves (Lm) and petioles (P)) and fruits in four developmental stages were sampled in three biological replicates (three separate plants). The four developmental fruit stages were: small green (sGF) (length 23.08 ± 1.77 (SD) mm (*n* = 6)), large green (lGF) (length 34.52 ± 4.44 (SD) mm (*n* = 6)), white (WF) (before turning stage) and red (RF) (ripe). Leaf and petiole samples were cut off with a sharp scalpel and were frozen immediately in liquid nitrogen. Fruits, receptacle with achenes, were cut into small pieces after removal of the calyx and rapidly frozen in liquid nitrogen. For detailed RNA extraction and reverse transcription protocols, please refer to Supplementary file 1.

An early season *F. x ananassa* cultivar, Cléry, and a midseason cultivar, Elsanta, were used in this expression study. For details on the sampling location and growing practices, please refer to Supplementary file 1. All samples were taken between 1.5 and 3.5 h after sunrise. All Cléry samples (young and mature leaves, petioles and four fruit developmental stages) were harvested on 1 April 2015. Elsanta young and mature leaves, petioles, white and red fruits were collected on 21 April 21 2015. Due to bad RNA quality (see below) Elsanta small and large green fruit samples from 21 April were discarded and resampled on 22 May 2015, from different plants than those sampled on 21 April. The second sampling is referred to as biological replicates 4, 5 and 6.

### RT-qPCR

**Fig. 1:**
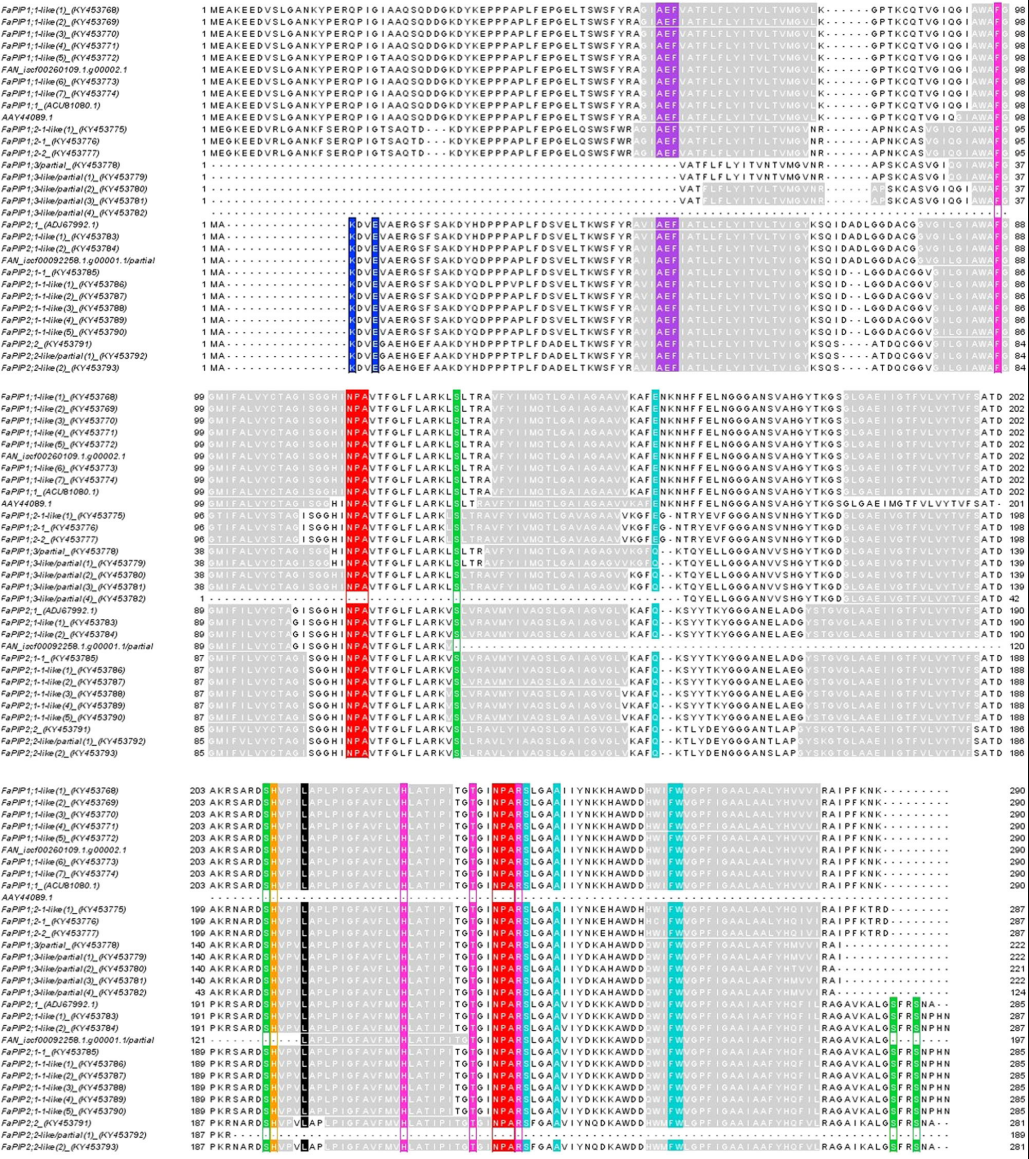
Alignment of amino acid sequences of *Fragaria x ananassa* PIP aquaporins Transmembrane domains (TM): grey; NPA motif: red; AEF motif: purple; Ar/R selectivity filter (FHTR): pink; P1–P5 residues: turquoise; putative conserved phosphorylation sites (S): green; putative conserved methylation sites (K and E): blue; putative conserved protonation site (H): orange; putative conserved blocking residue (L): black

**Fig. 2:**
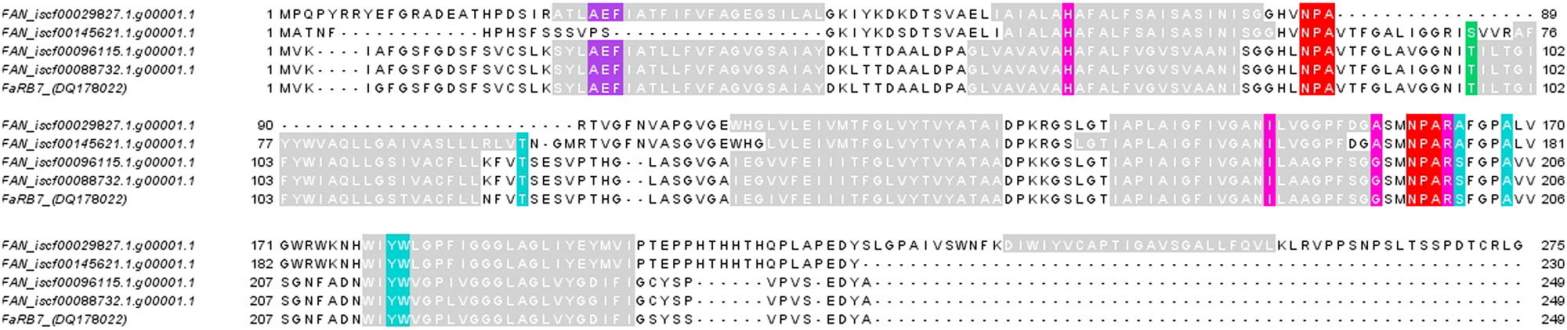
Alignment of amino acid sequences of *Fragaria x ananassa* TIP aquaporins Transmembrane domains (TM): grey; NPA motif: red; AEF motif: purple; Ar/R selectivity filter (HIA/GR): pink; P1–P5 residues: turquoise; putative conserved phosphorylation sites (S/T): green

All obtained aquaporin coding sequences were aligned (Figs. 1 and 2) and divided into eight different groups based on the visual interpretation of multiple sequence alignments and sequence similarities in the untranslated regions. Using Primer3Plus (http://primer3plus.com/cgi-bin/dev/primer3plus.cgi), primers were designed in such a way that they amplified all sequences within one group but not the sequences belonging to other groups. In Table 1, the aquaporin sequences belonging to each group are listed. Gene specific amplification efficiencies were determined by LinRegPCR (Table 1)^37,38^. Based on geNorm analyses, *clathrin* and *CHP3* were selected as reference genes^39^. Both reference genes had amplification efficiencies of 1.911. For detailed RT-qPCR protocols, please refer to Supplementary file 1.

**Table 1:** Grouping of aquaporin sequences based on sequence similarities and RT-qPCR primers for amplification of each group of aquaporins with the corresponding amplification efficiencies

Group	(Partial) Aquaporins	Genbank acc. no.	Forward primer (F) (5’–3’) Reverse primer (R) (5’–3’)	Amplification efficiency
FaPIP1;1	FaPIP1;1 partial *F. x ananassa* PIP FaPIP1;1-like (1-7) FAN_iscf00260109.1.g00002.1	GQ390798.1 (ref. ^30^) DQ022749.1 (ref. ^35^) KY453768–KY453774	(F) CTTGGGAGCCAACAAGTACC (R) TCCTTGTAGTCCTTGCCGTC	1.895
FaPIP1;2	FaPIP1;2-1(-like(1)) FaPIP1;2-2	KY453775–KY453776 KY453777	(F) GACCTCAGCTCAGACTGACA (R) CTCTCCAGAAAGACCATGACTG	1.902
FaPIP1;3	FaPIP1;3/partial FaPIP1;3-like/partial(1-4)	KY453778 KY453779–KY453782	(F) AGCAAGGAAGCTCTCACTCA (R) GTGTAGCCATGACTCACAACG	1.881
FaPIP2;1 (a)	FaPIP2;1 FaPIP2;1-like(1-2) FAN_iscf00092258.1.g00001.1/partial	GQ390799.1 (ref. ^31^) KY453783–KY453784	(F) CAAGTCCCAAATCGACGCC (R) ATGTGACCCCCTGAGATTCC	1.786
FaPIP2;1 (b)	FaPIP2;1-1(-like(1-5))	KY453785–KY453790	(F) CTCCGCTCTTCGACTCAGTT (R) CCTCCGAGGTCGATTTGGG	1.902
FaPIP2;2	FaPIP2;2 FaPIP2;2-like/partial(1) FaPIP2;2-like(2)	KY453791 KY453792 KY453793	(F) TACAAGAGCCAAAGCGCAAC (R) AGCTGGGTTGATGTGTCCTC	1.897
FaTIP (a)	FAN_iscf00029827.1.g00001.1 FAN_iscf00145621.1.g00001.1		(F) TATAGGGGGTGGATTGGCAG (R) TAGTAGTCCTCGGGAGCCAA	2.081
FaTIP (b)	FaRB7 FAN_iscf00096115.1.g00001.1 FAN_iscf00088732.1.g00001.1	DQ178022.1 (ref. ^29^)	(F) CTCCGGTGGCCATTTGAAC (R) ACACTCTCGCTGGTGACAAA	1.903

### Statistical analysis

RT-qPCR data were analysed using SPSS (version 2.2). Relative expression values were log-transformed. Because of the low number of replicates, the Brown–Forsythe test (robust test of equality of means) was used in combination with the Scheffé post hoc test at the 5% significance level. For an overview of all tests performed, please refer to Supplementary file 1.

## Results

### Identification and analysis of aquaporin coding sequences

Five fragments of *F. x ananassa* coding sequences were obtained by RT-PCR using primers designed on *F. vesca* PIP-coding sequences and one partial *F. x ananassa* coding sequence (Genbank Acc. No. DQ022749.1) (Table S3)^28,35^. In addition to these fragments, one *F. x ananassa* EST (Genbank Acc. No. GW403182.1) resulted from a BLAST using the FaPIP1;1 (Genbank Acc. No. GQ390798.1) coding sequence as a query. By means of RACE PCR, the coding sequence of these fragments was extended towards both the 5’ and 3’ ends of the coding sequences. The start codon and/or stop codon could not be found for all sequences (Fig. 1). Using the obtained sequences, primers were designed for amplification of full or partial coding sequences of aquaporins from *F. x ananassa* cv. Elsanta and Diamante cDNA. After ligation of the full or partial coding sequence into a vector and amplification in *E. coli* several positive colonies per amplification reaction were sequenced on both strands. Because of the presence of eight alleles in each *F. x ananassa* cultivar, several sequences that slightly differ from each other were amplified by the same primers (Table S5). Additionally, six coding sequences were derived from the Strawberry GARDEN database using the BLAST tool: FAN_iscf00260109.1.g00002.1; FAN_iscf00092258.1.g00001.1/partial; FAN_iscf00029827.1.g00001.1; FAN_iscf00145621.1.g00001.1; FAN_iscf00096115.1.g00001.1; FAN_iscf00088732.1.g00001.1 (Supplementary file 2). To classify the new aquaporins, a phylogenetic analysis, including aquaporins from *Fragaria vesca*, *Malus domestica*, *Vitis vinifera* and *Arabidopsis thaliana* was conducted (Supplementary file 3). This clearly identifies the new aquaporins as PIP subtype 1, PIP subtype 2 or TIP aquaporins. Based on sequence similarities within the coding sequence and similarities in the 5’ and 3’ UTR, several coding sequences were named as alleles from the same gene. Sequence names were chosen to reflect the partitioning into different groups according to the phylogenetic tree (Supplementary file 3) and to reflect the similarities in coding sequence and UTR regions (Supplementary file 4).

For all full coding sequences except FAN_iscf00145621.1.g00001.1, six transmembrane domains were predicted (Figs. 1 and 2). This is in accordance with the properties of other aquaporins^17,40^.

Next, the presence of conserved residues among all or specific (sub)classes of aquaporins (Table S6) was confirmed in all sequences presented here (Figs. 1 and 2), except for the Ser/Thr in the B-loop of FAN_iscf0029827.1.g00001.1. NPA motifs are conserved among all classes of aquaporins, unlike the residues making up the Ar/R selectivity filter, which can differ between and even within subclasses. While PIP Ar/R filters are always made up by F, H, T and R, the TIPs carry a high diversity in these residues, among others the combinations found here (HIAR and HIGR)^8,18^. The position of the conserved Leu residue was described by Törnroth-Horsefield et al. to be in loop D^23^, but the corresponding residues in the sequences presented here are predicted by the TMHMM software to be lying in the transmembrane helix following loop D. Since this L residue is highly conserved and its location in SoPIP2;1 is based on the X-ray structure of the protein, localization in loop D can be considered as the true location^23^. The phosphorylation site in loop B in TIPs is part of a RXSXXR motif in most α-TIPs and of a TXXR motif in δ-TIPs^41^. This motif provides an indication that FAN_iscf00145621.1.g00001.1 is an α-TIP. The other TIP cds’s possess only the conserved Thr, the surrounding residues do not match these motifs, indicating they belong to another type. FAN_iscf29827.1.g0000.1 lacks the phosphorylation site itself, but the other conserved residues are present in this cds, as well as six transmembrane domains, providing still enough evidence that this sequence is a TIP.

Consequently, indications of aquaporin water permeability were obtained from a residue in transmembrane domain 2 (TM2) and one in loop E (positions 103 and 249, respectively, in FaPIP1;1-like(1)_(KY453768) in Fig. 1)^42^. In TM2, Ala is found in all PIP1 sequences, Ile/Val in all PIP2. At the position in loop E, Ile is present in all PIP1 sequences, Val in PIP2s. These residues correspond to lower predicted water permeability for PIP1s and higher predicted water permeability for PIP2s^42–44^.

Thereafter, the residues at the positions of the SSSSs and putative SDPs were retrieved from the cds’s (Tables 2 and 3)^8,26^. These residues were compared to the SSSSs and SDPs suggested in literature for each substrate and the corresponding substrates were listed.

**Table 2:** SSSS residues in the sequences presented in this study and substrates matching those residues. Group members are listed in Table 1

Group	NPA consensus sequence		Ar/R selectivity filter				P1–P5					Residue at conserved L in loop D^a^	Substrates according to Hove et al. (ref. ^8^)	Substrates according to Azad et al. (ref. ^26^)
	Loop B	Loop E	TM 2	TM 5	Loop E (1)	Loop E (2)	P1	P2	P3	P4	P5			
FaPIP1;1	SGGHI**NPA**VT	GTGI**NPA**RSLG	F	H	T	R	E	S	A	F	W	L	–	–
FaPIP1;2	SGGHI**NPA**VT	GTGI**NPA**RSLG	F	H	T	R	E	S	A	F	W	L	–	–
FaPIP1;3	SGGHI**NPA**VT	GTGI**NPA**RSLG	F	H	T	R	Q	S	A	F	W	L	H_2_O_2_, urea, boron, CO_2_	H_2_O_2_, CO_2_
FaPIP2;1 (a)	SGGHI**NPA**VT	GTGI**NPA**RSLG	F	H	T	R	Q	S	A	F	W	L	H_2_O_2_, urea, boron, CO_2_	H_2_O_2_, CO_2_
FaPIP2;1 (b)	SGGHI**NPA**VT	GTGI**NPA**RSLG	F	H	T	R	Q	S	A	F	W	L	H_2_O_2_, urea, boron, CO_2_	H_2_O_2_, CO_2_
FaPIP2;2	SGGHI**NPA**VT	GTGI**NPA**RSFG	F	H	T	R	Q	S	A	F	W	L	H_2_O_2_, urea	H_2_O_2_
FaTIP (a)	SGGHV**NPA**VT^b^	GASM**NPA**RAFG	H	I	A	R	T	A	A	Y	W	I	H_2_O_2_, urea	
FaTIP (b)	SGGHL**NPA**VT	GGSM**NPA**RSFG	H	I	G	R	T	S	A	Y	W	I	H_2_O_2_, urea, ammonia	H_2_O_2_, urea, ammonia

^a^Table S6, Figs. 1 and 2

^b^FAN_iscf00029827.1.g00001.1 does not correspond to this SSSS due to a gap in the alignment starting immediately after NPA. Bold values indicate conserved NPA motifs.

**Table 3:** Residues in the sequences presented in this study at the putative specificity-determining positions (SDPs) suggested by Hove et al.^8^ Residues that do not match the ones listed by Hove et al. are highlighted in black. Group members are listed in Table 1. The SDPs that are also part of an NPA motif or are one of the Froger’s positions (P1–P5) are shown in bold

Group	Boric Acid		CO_2_		H_2_O_2_		Urea		Substrates matching SDPs according to Hove et al. (ref. ^8^)
	Position^a^	SDP residue	Position^a^	SDP residue	Position^a^	SDP residue	Position^a^	SDP residue	
FaPIP1;1	108	T	136	V	146	A	115	H	H_2_O_2_ Urea Boric acid
	111	I	139	I	149	G	118	**P**	
	115	H	143	T	153	V	122	F	
	118	**P**	146	A	156	F	125	F	
	186	E	214	I	220	I	193	L	
	226	L	254	K	260	H	233	P	
	229	L	257	W	263	**F**	236	G	
	231	T	259	D	265	V	238	G	
	233	P	261	W	267	P	240	**N**	
FaPIP1;2	108	T	136	V	146	A	115	H	H_2_O_2_ Urea Boric acid
	111	I	139	I	149	G	118	**P**	
	115	H	143	T	153	V	122	F	
	118	**P**	146	A	156	F	125	F	
	186	E	214	I	220	I	193	L	
	226	L	254	E	260	H	233	P	
	229	L	257	W	263	**F**	236	G	
	231	T	259	H	265	V	238	G	
	233	P	261	W/C	267	P	240	**N**	
FaPIP1;3	108	T	136	V	146	A	115	H	H_2_O_2_ Urea Boric acid
	111	I	139	I	149	G	118	**P**	
	115	H	143	T	153	V	122	F	
	118	**P**	146	A	156	F	125	F	
	186	E	214	I/V	220	I	193	L	
	226	L	254	A	260	Q	233	P	
	229	L	257	W	263	**F**	236	G	
	231	T	259	D	265	V	238	G	
	233	P	261	W	267	P	240	**N**	
FaPIP2;1 (a)	108	T	136	V	146	A	115	H	H_2_O_2_ Urea
	111	I	139	I	149	G	118	**P**	
	115	H	143	S	153	V	122	F	
	118	**P**	146	A	156	F	125	F	
	186	E	214	V	220	I	193	L	
	226	M	254	K	260	Q	233	P	
	229	L	257	W	263	**F**	236	G	
	231	T	259	D	265	V	238	G	
	233	P	261	W	267	P	240	**N**	
FaPIP2;1 (b)	108	T	136	V	146	A	115	H	H_2_O_2_ Urea
	111	I	139	I	149	G	118	**P**	
	115	H	143	S	153	V	122	F	
	118	**P**	146	A	156	F	125	F	
	186	E	214	V	220	I	193	L	
	226	M	254	K	260	Q	233	P	
	229	L	257	W	263	**F**	236	G	
	231	T	259	D	265	V	238	G	
	233	P	261	W	267	P	240	**N**	
FaPIP2;2	108	T	136	V	146	A	115	H	Urea
	111	I	139	I	149	G	118	**P**	
	115	H	143	S	153	V	122	F	
	118	**P**	146	A	156	F	125	F	
	186	E	214	V	220	I	193	L	
	226	M	254	D	260	H	233	P	
	229	L	257	W	263	**F**	236	G	
	231	T	259	D	265	L	238	G	
	233	P	261	W	267	P	240	**N**	
FaTIP (a)	No TIPs		No TIPs		116	A	85	H	Urea
					119	A	88	**P**	
					123	L	92	F	
					126	V	95	L	
					182	I	158	L	
					219	H	195	P	
					222	**Y**	198	G	
					224	L	200	S	
					226	P	202	**N**	
FaTIP (b)	No TIPs		No TIPs		116	S	85	H	Urea
					119	A	88	**P**	
					123	L	92	F	
					126	V	95	A	
					182	I	158	L	
					219	N	195	P	
					222	**Y**	198	G	
					224	V	200	S	
					226	P	202	**N**	

^a^Position is relative to the numbering used in Fig. 1 for FaPIP1;1-like(1)_(KY453768) for all PIP groups and relative to the numbering used in Fig. 2 for both TIP groups

Sequences belonging to groups FaPIP1;1 and FaPIP1;2 have an E residue in position P1, which does not occur in any of the SSSSs suggested by Hove et al. or Azad et al.^8,26^. Apart from this P1 position, groups FaPIP1;1 and FaPIP1;2 have the same SSSS residues as groups FaPIP1;3, FaPIP2;1(a) and FaPIP2;1(b), pointing at transport of boron, CO_2_, H_2_O_2_ and urea according to Hove et al.^8^. Group FaPIP2;2 has the SSSS residues for transport of H_2_O_2_ and urea, but one residue in the loop E NPA signature differs from that for boron and CO_2_. Azad et al., contrary to Hove et al., provide no boron SSSS for PIPs or TIPs and no urea SSSS for PIPs^8,26^. Group FaTIP(a) is predicted to transport H_2_O_2_ and urea, as does group FaTIP(b), that is, additionally, also predicted to transport ammonia according to Hove et al.^8^. The SSSSs for H_2_O_2_ and urea are slightly different according to Azad et al. and don’t match the residues in group FaTIP(a)^26^. Silicon SSSSs don’t match since silicon transport appears to be unique for NIPs^8,26,42^.

Finally, SDP residues were listed for the sequences presented here (Table 3). These SDPs are also proposed by Hove et al., in addition to the SSSSs^8^. Ammonia SDPs are exclusively listed for TIPs, but the residues do not match those in the TIPs presented here and are therefore omitted from Table 3. No TIP residues were listed for boric acid and CO_2_. All PIP1 groups have SDP residues matching those listed by Hove et al. for boric acid, H_2_O_2_ and urea^8^. FaPIP2;1 groups only match the SDPs listed for H_2_O_2_ and urea and group FaPIP2;2 and both TIP groups only match those for urea. In some cases, only one out of nine residues does not match the ones listed by Hove et al.^8^. This is the case for boric acid transport by both FaPIP2;1 groups, for boric acid, CO_2_ and H_2_O_2_ transport by FaPIP2;2 and for H_2_O_2_ transport by TIPs.

### Tissue-specific expression analysis

**Fig. 3:**
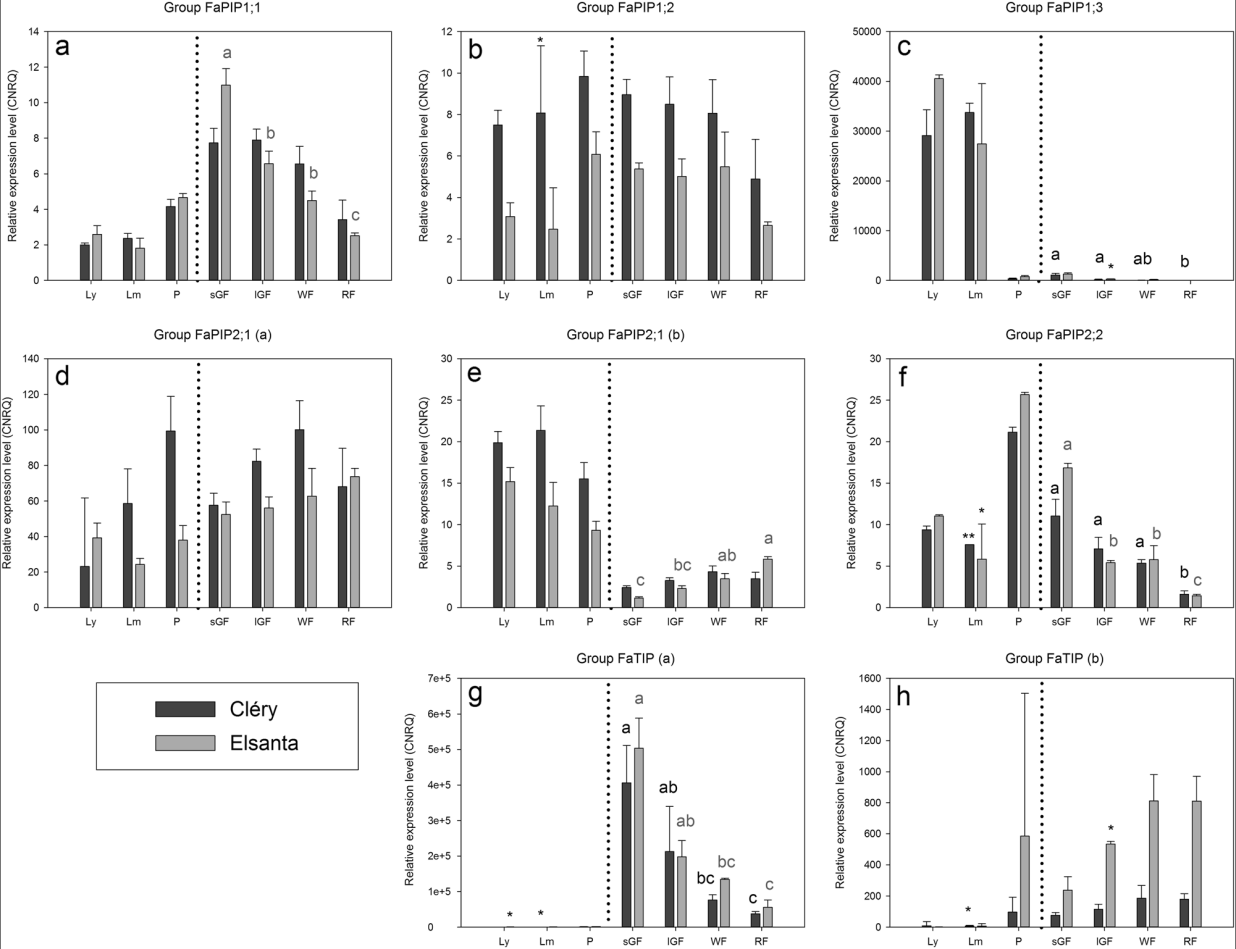
Relative expression levels (non-log-transformed CNRQ) per gene group across different tissues (**a**) group FaPIP1;1 (**b**) group FaPIP1;2 (**c**) group FaPIP1;3 (**d**) group FaPIP2;1(a) (**e**) group FaPIP2;1(b) (**f**) group FaPIP2;2 (**g**) group FaTIP(a) (**h**) group FaTIP(b) Ly young leaf, Lm mature leaf, P petiole, sGF small green fruit, lGF large green fruit, WF white fruit, RF red fruit. Geometric means of 3 biological replicates ± standard error. When the difference in Cq value between noRT and sample was smaller than 5, this sample was left out of the dataset, resulting in some mean relative expression levels based on 2(*) or 1(**) biological replicate(s). Letters indicate statistically significant differences between fruit developmental stages (*p* < 0.05)

The newly obtained sequences described above, together with some coding sequences derived from the strawberry GARDEN database and the *F. x ananassa* (partial) PIP aquaporin coding sequences that had already been described in literature, were divided into eight groups (Table 1)^29–31^. RT-qPCR was used to determine the expression level of each group across different tissues. The quality of the DNase treated RNA samples was shown to be good for all samples used in RT-qPCR, i.e. all Cléry samples, Elsanta young and mature leaf, petiole, white and red fruit replicates 1, 2 and 3 and Elsanta small and large green fruit replicates 4, 5 and 6. Statistical tests were performed to compare the expression between different tissues and groups of tissues for each aquaporin group. Significant differences between fruit developmental stages are indicated in Fig. 3.

The expression patterns are generally the same for both the early season (Cléry) and the midseason cultivar (Elsanta) (Fig. 3). Only in some tissues and for some aquaporin groups, there are significant expression differences between cultivars (Table S7).

Group FaPIP1;1 (Fig. 3a) shows a gradual downregulation of the expression during fruit development. Only in Elsanta, this downregulation is significant (*p* < 0.001). The expression of this group in leaves and petioles is significantly smaller than in the fruit tissues, but still substantial (*p* < 0.001 and *p* = 0.015 for Cléry and Elsanta, respectively).

Group FaPIP1;2 (Fig. 3b) seems to be ubiquitously expressed under the conditions of this study, no significant differences could be found between the different tissues.

Group FaPIP1;3 (Fig. 3c) is nearly leaf-specific, the difference between leaf and other tissues is significant (*p* < 0.001, both cultivars). The modest expression in the fruits is significantly downregulated during development and ripening in Cléry (*p* = 0.028).

Both groups FaPIP2;1 (a) and (b) (Fig. 3d,e) are upregulated during fruit development and ripening (except for group FaPIP2;1(b) in Cléry). This trend is only significant for group FaPIP2;1(b) in Elsanta (*p* = 0.002). The expression of group FaPIP2;1 (a) (Fig. 3d) is higher in fruit tissue when compared to the expression in leaves and petioles in Elsanta (*p* = 0.001). Group FaPIP2;1 (b) (Fig. 3e) on the other hand is predominantly expressed in vegetative aboveground tissues (*p* < 0.001, both cultivars).

Group FaPIP2;2 (Fig. 3f) shows an expression pattern similar to group FaPIP1;1 (Fig. 3a), namely downregulation during fruit development (*p* = 0.002, Cléry; *p* = 0.003, Elsanta). Expression of this group in leaves and petioles is significantly higher than in fruit tissue (*p* = 0.005, Cléry; *p* = 0.027, Elsanta).

For group FaTIP (a) (Fig. 3g) expression in the leaves and petioles is negligible compared to the fruits (*p* < 0.001, both cultivars). This group of aquaporins is also downregulated during fruit development and ripening (*p* = 0.015, Cléry; *p* = 0.010, Elsanta).

For FaTIP (b) (Fig. 3h) upregulation during fruit development is significant for Elsanta (*p* = 0.046), but post hoc tests do not indicate differences. There is no significant variation in FaTIP(b) expression in Cléry (Fig. 3h). Expression in leaves is markedly lower compared to other tissues (i.e. petioles and fruits) (*p* = 0.014, Cléry; *p* < 0.001, Elsanta).

## Discussion

### Gating, subcellular localization and water permeability

The presence of certain conserved residues that have been attributed a function in literature provides us with information regarding the mechanisms that are potentially involved in gating (opening/closure of the water channel) or subcellular localization of the aquaporins presented here. Likewise, other conserved residues indicate that in *Fragaria* PIP1 aquaporins have a lower water permeability than PIP2 aquaporins. This phenomenon has been widely described in literature^12^.

### Substrate specificity

Several residues can be used to predict substrate specificity of aquaporins^26,42,45^. SSSSs at the NPA and Ar/R filters and at the Froger’s P1–P5 positions have been suggested per substrate, along with additional SDPs) outside of these filters (Tables 2 and 3)^8,26^.

The only group carrying the suggested SSSS for ammonium is group TIP(b), which is in accordance with experimental evidence^25,26,42^.

H_2_O_2_ transport is predicted for groups FaPIP1;3, FaPIP1;2 (a) and (b) by Hove et al. and Azad et al.^8,26^. Azad et al. suggest different SSSSs for H_2_O_2_ in TIPs than Hove et al. do, excluding group FaTIP(a) from the H_2_O_2_ transporters^8,26^. These results deviate from what is seen in barley, where H_2_O_2_ transport is predicted to be restricted to TIPs. Transport assays show that several PIP2s and TIPs transport H_2_O_2_, but tested PIP1s were never shown to transport this solute^8,25^.

When considering the SSSS only, CO_2_ is likely to be transported by groups FaPIP1;3, FaPIP2;1(a) and (b) (Table 2). When also considering the SDP suggested by Hove et al., more than one SDP residue deviates (Table 3)^8^. Functional tests will have to elucidate whether CO_2_ is transported by these aquaporins or not, validating or contradicting the suggested SDPs. Experimental evidence for CO_2_ transport exists (only) for PIPs, but very few aquaporins have been tested^8,25^.

Regarding boron and urea transport, SSSSs from Azad et al. do not comply with Hove et al.^8,26^. Hove et al. include PIPs experimentally proven to transport boron and urea in their analysis, while Azad et al. base their analysis on a selection made by Perez Di Giorgio et al., not listing PIPs^8,25,26^. Discrepancies between both selections apparently lead to different SSSS outcomes.

According to Hove et al., boron is predicted to be transported by group FaPIP1;3 (Table 2)^8^. Azad et al., however, don’t provide an SSSS for PIPs^26^. In literature, boron has been experimentally proven to be transported by PIP1s, but generally boron transport seems to be a feature of NIPs rather than PIPs^3,8,25^.

Urea is predicted to be transported by all of the sequences presented here, except for groups FaPIP1;1 and FaPIP2;1. Both the SSSS and SDPs correspond to Hove et al. (Tables 2 and 3)^8^. According to Azad et al., however, only group FaTIP(b) transports urea. Experimental evidence exists for urea transport by TIPs, studies providing evidence for urea transport by PIPs are less numerous^8,25,26^.

In summary, groups FaPIP1;3, FaPIP2;1(a) and (b) are predicted to transport H_2_O_2_. Group FaTIP(b) is predicted to transport ammonia and urea. These predictions are based on full correspondence to all SSSSs and SDPs, both according to Hove et al. and Azad et al.^8,26^. When considering the SSSSs and SDPs suggested by Hove et al. only, groups FaPIP1;3, FaPIP2;1(a) and (b), FaPIP2;2 and FaTIP(a) transport urea too and group FaPIP1;3 transports boron^8^. Since all substrates discussed here have important physiological functions (reviewed in refs. ^8,26^), it’s worthwhile having an idea of which aquaporins can transport them. We must remark that validation of these predictions through in vivo tests is still required.

### Tissue-specific expression analysis

Our results clearly show that PIP1 aquaporins are substantially expressed in Cléry and Elsanta leaves and for two out of three PIP1 groups also in petioles (Fig. 3). The expression of the FaPIP1;1 aquaporin in leaves has also been investigated in cultivars Selva and Camarosa by Northern blot^30^. The probe is considered general for all PIP1^31^. In contrast to the RT-qPCR results presented here, expression in leaves and petioles was not detected^30^. Cultivar differences cannot be ruled out, but this discrepancy could also be explained by the fact that the primers amplifying group PIP1 detect a pool of mRNA’s, showing the combined expression levels of several aquaporins. As the probe used for the Northern blot analysis spans the entire coding sequence, it is potentially more specific and shows the (combined) expression levels of only one or a subset of the genes considered here in group FaPIP1. Another possible explanation is that the sampling time causes this difference in expression. It was shown that PIPs exhibit a diurnal expression pattern in *F. vesca* leaves, with a peak about 2 h after sunrise and an up to 14 fold lower expression in the late afternoon^28^. It is possible that, at the time of sampling (which is not mentioned), expression in the Selva and Camarosa samples had dropped to levels no longer detectable by Northern blot.

The expression data presented here for fruits is the combined level of receptacle and achene expression. Since the water uptake/release is much smaller in achenes, the major water movements take place in the receptacle. Consequently, variation in aquaporin expression in achenes is likely to be minimal compared to that in the receptacle. We can thus argue that the majority of the variation in expression will originate from the receptacle. This assumption must, however, be confirmed by RT-qPCR analysis on receptacle tissue only.

We demonstrated downregulation of group FaPIP1;1 during ripening. In Elsanta, significantly lower expression was found for red fruits compared to green fruits. The expression of FaPIP1;1 was also investigated in different developmental stages of fruits in the cultivars Selva and Camarosa, using Northern blotting. In contrast to our RT-qPCR data, expression was shown to increase during fruit ripening^30^. Neither group FaPIP1;1, nor one of the two other PIP1 groups show a pattern consistent with this^30^. Upregulation was also found in cultivars Toyonaka and Camarosa with a probe spanning the entire open reading frame^31^.

For groups FaPIP2;1(a) and FaPIP2;1(b), there is an increase in expression from the large green to the white stadium and a decrease from white to red for Cléry. For Elsanta, expression is rather stable from the green to the white stage, but shows an increase towards the red stage. The same Northern blot analysis as described above for FaPIP2;1 was performed and the expression pattern was found to increase from the green to the white stage and to decrease again towards the red stage in Camarosa, although this decrease is smaller than our RT-qPCR data show for Cléry^31^. For Toyonaka, the response was not pronounced, the expression seems to be rather stable. The patterns during fruit development and ripening found in Cléry and Elsanta are comparable to the ones found in Camarosa and Toyonaka respectively.

In three PIP groups we observe significant downregulation during fruit development (group FaPIP1;1 (Elsanta), group FaPIP1;3 (Cléry) and group FaPIP2;2 (Elsanta and Cléry)), while upregulation at least until the white stage is displayed for two PIP groups (FaPIP2;1(a) and FaPIP2;1(b) (significant for Elsanta)). In *Vitis vinifera*, a majority of the PIP genes was downregulated during fruit ripening, also in seeded berries^46–48^. In seeded tomato fruits (*Solanum lycopersicum* Micro-Tom), the majority of PIPs tested also showed a downregulation from the green towards the red stadium (SlPIP1;2, SlPIP1;7, SlPIP2;1, SlPIP2;4, SlPIP2;8 and SlPIP2;9)^49^. Other tomato research shows a diversity in expression patterns for eight PIPs throughout fruit development. Some are downregulated during fruit development, some show a higher expression in the turning stage than in the green or red stage^50^, which is similar to the patterns found here in groups FaPIP2;1(a) and FaPIP2;1(b) for strawberry. High expression in the pré-veraison stage of grapevine berries was also reported for eight PIP transcripts, only three of which were induced during ripening^51^. Four PIP ESTs, belonging to two different PIP genes according to the authors, were also downregulated during parthenocarp Clementina mandarin ripening^52^.

Up to 10 days after anthesis, cells in the developing strawberry fruit divide. From 10 till 20 days after anthesis, fruits grow and this is only due to cell enlargement by water uptake^53^. This period of strong increase in cell volume reaches from the small green to the white fruit stadium. During the periods of fruit volume increase, a lot of water needs to be transported from the fruit vascular system towards the peripheral parenchyma. A high PIP expression level could aid in this water distribution. During periods of slower growth, water demand by peripheral tissues drops and so does the PIP expression. This could explain the downregulation of several PIPs during fruit development. In grapevine, expression of PIPs was shown to coincide with the periods of berry growth, while during the periods of slower growth, PIP expression was downregulated^48^. Also in apple (*Malus domestica*) it has been reported that expression of one PIP coincides with fruit cell expansion^54^. Cultivar Elsanta has a slightly different growth curve (double sigmoid)^55^. There are two periods of increase in cell and fruit volume: the strongest increase happens roughly between the small green and the large green fruit stadium (up to 15 days after anthesis), during the white stadium growth and cell expansion slow down. A second growth period occurs during the coloration of the fruit (about 25 to 30 days after anthesis)^56^. Consequently, a temporarily lower PIP expression during the white stage could be expected because of the sigmoid growth curve, but this is not reflected in our data. Our results can also point at a more dominant role for symplastic water transport in riper fruit, as was proposed for grapevine^52^. This route might be partially impaired in the immature fruit stages.

We observed no TIP expression in the leaves. Expression of FaRB7, classified in group FaTIP(b), has been investigated in *F. x ananassa* cv. Calypso by means of Northern analysis and reverse transcription PCR (RT-PCR)^29^. Our results confirm these data. Absence of expression of these two groups indicates that not yet identified TIPs must be responsible for regulating water influx and efflux to/from the vacuoles in leaves.

The significant downregulation of group FaTIP(a) during fruit development supports the hypothesis stated before. During the periods of fruit expansion a lot of water needs to be taken up by the vacuole to provide sufficient turgor for cell expansion. Later on, the water must be contained within the vacuole, causing FaTIP(a) levels to drop.

The expression of group FaTIP(b) found in red fruits in Elsanta and to lesser extent in Cléry could not be demonstrated in Calypso^29^, but our results also indicate that big differences in expression between cultivars within one tissue can exist for this group of TIPs. Also, the primers designed here probably amplify more transcripts than are detected by the probe used in Northern blot. FaTIP(b) is probably subject to a different regulatory mechanism than group FaTIP(a), supporting the theory of specialization of isoforms within one subgroup^52^.

Downregulation of TIPs during fruit ripening was also demonstrated in seeded *Vitis vinifera* berries and for some TIPs in seeded tomato fruits (*Solanum lycopersicum* Micro-TOM) (SlTIP1;1, SlTIP2;1, SlTIP3;1)^46,49^. In parthenocarp clementine mandarins, a δ-TIP was downregulated while a γ-TIP was upregulated^52^.

### Aquaporin functions suggested by predicted transport and expression profiles

Combining predicted substrate specificity and expression profiles, hypothesis can be formulated about the function of each group of aquaporins. Hereby, one must keep in mind that for *Fragaria* and many other species, it has been shown that PIP1 type aquaporins have a limited intrinsic water permeability, but they greatly enhance the water permeability of PIP2 type aquaporins^30,31^. Co-expression with a PIP2 is needed for PIP1s to reach the plasma membrane^15^. This implies that individual expression patterns do not show the full picture, since heteromerization defines the net effect on water permeability.

Groups FaPIP1;1 and FaPIP2;2 are clearly downregulated during fruit ripening. Taken together with the fact that these groups are predicted to transport only water, this indicates that their main function lies in regulating the water balance in the rapidly expanding and soluble sugar accumulating, ripening fruit. Expression in non-fruit tissues indicates that also in vegetative tissue, this group of aquaporins is involved in regulating cell-to-cell water transport.

Group FaPIP1;2 is constitutively expressed under commercial greenhouse circumstances and not predicted to transport non-aqua substances. This could point at a role in supporting the basal cell metabolism.

Group FaPIP1;3 seems to be leaf-specific, suggesting a dedicated role in leaf cells (no data available on root expression). If they would be located to the thylakoid membrane, they might aid in thylakoid lumen filling^3^. The combination of leaf-specific expression and predicted H_2_O_2_ transport could indicate a function in ROS dissipation in chloroplasts, provided it is located in the chloroplast envelope^57^. Leaf-specific expression can also point at a function in CO_2_ import towards the chloroplasts^26^. However, there are no predicted CO_2_ transporters among the aquaporins presented here.

Groups FaPIP2;1(a) and (b) are expressed in all tested tissues and upregulated during fruit ripening. Taken together with the high water permeability that is common for PIP2 type aquaporins, these aquaporins are expected to sustain water flows needed for basal cell metabolism, also in fruits. Upregulation or downregulation of these aquaporins could adapt the water flows consecutive stages of fruit ripening and potentially to changing environmental conditions. These aquaporins are also predicted to transport H_2_O_2_, which might be indicative of a role in cell signalling or ROS dissipation.

Fruit-specific expression (no data available on root expression) of group FaTIP(a) (downregulation during ripening) and no predicted non-water substrates strongly suggest that this group of aquaporins is involved in regulating water flows to build up turgor in expanding fruit cells upon accumulation of soluble sugars.

Group FaTIP(b) is not expressed in leaves and upregulated during fruit ripening in only one of two cultivars. This group is predicted to transport ammonia and urea, indicating a role in nitrogen acquisition and balance. However, other, more regulated transport systems for ammonia and urea are present in the tonoplast, suggesting this role is minor^3^.

In this study, we present a number of new *F. x ananassa* aquaporins, belonging to different (sub)classes (PIP1, PIP2 and TIP). These coding sequences will contribute to the extension of our understanding of the regulation of water transport at the cellular level in plants. Presence of conserved residues, predicted substrate specificity and expression patterns are indicative of the aquaporin functions. Our findings confirm functional specialization among aquaporins, even within the same (sub)class. Now these sequences are available, their function in regulating plant–water relations can be further investigated.

## Electronic supplementary material

Supplementary file 1

Supplementary file 2

Supplementary file 3

Supplementary file 4

Supplementary figures

Supplementary tables
