# Supplementary material for: Identification and substrate prediction of new Fragaria x ananassa aquaporins and expression in different tissues and during strawberry fruit development
Source: Hortic Res. 2018 Apr 1;5:20. doi: 10.1038/s41438-018-0019-0 (PMC5880810; doi:10.1038/s41438-018-0019-0)
Supplement: Supplementary file 1 [file 41438_2018_19_MOESM1_ESM.docx]

**Article title**: Identification and substrate prediction of new *Fragaria x ananassa* aquaporins and expression in different tissues and during strawberry fruit development

**Journal**: Horticulture Research

**Authors**: Britt Merlaen, Ellen De Keyser and Marie-Christine Van Labeke

**Corresponding author**: Marie-Christine Van Labeke, Plant Production, Faculty of Bioscience Engineering, Ghent University, Coupure Links 653, 9000 Gent, Belgium Email: mariechristine.vanlabeke@ugent.be

**Supplementary file 1** Detailed protocols and supplementary information to Materials and Methods; Overview of statistical tests performed; References to this file

## RNA extraction and reverse transcription

*Fragaria x ananassa* tissues were sampled, frozen in liquid nitrogen and ground using an IKA A11 basic analytical mill. The ground tissue was stored at -80 °C until RNA extraction. Five mL of hot (65 °C) extraction buffer (2 % CTAB, 2 % PVP (K40), 100 mM Tris-Base, 25 mM EDTA, 2.0 M NaCl, 0.5 gL^-1^ spermidine, 2 vol% 2- mercaptoethanol) was mixed with about 2 grams of ground tissue and incubated for at least 10 min at 65 °C. An equal volume of chloroform:isoamyl alcohol (24:1) was added and the samples were homogenized with a Beadbeater (B. Braun Biotech International GmbH, Melsungen, Germany) until a homogenous suspension was obtained (1 or 2 minutes) and centrifuged at room temperature for 10 min at 8 000 x g. An equal volume of chloroform:isoamyl alcohol (24:1) was added to the supernatant. The samples were vortexed for 30 seconds and centrifuged for 10 min at 8 000 x g at room temperature. LiCl was added to the supernatant to a final concentration of 2 M. The RNA was precipitated overnight in an ice bucket in a 4 °C fridge. The RNA was pelleted at 4 °C for 30 min at 10 000 x g. The pellet was resuspended in 500 µl SSTE (1M NaCl, 0.5 % SDS, 10 mM Tris pH 8, 1 mM EDTA). The RNA was extracted by adding 500 µl chloroform:isoamyl alcohol (24:1), vortexing and centrifuging for 10 min at 4 °C and 8 000 x g. The supernatant was transferred to a 2 mL tube and at least 2 volumes of ice cold 100% ethanol were added. The RNA was precipitated at -80 °C for 2 hours and pelleted at 4 °C for 30 min at 10 000 x g. The supernatant was decanted and 500 µl of 76 % ethanol, 0.3M NaOAc was added. After vortexing, the samples were centrifuged for 10 min at 4 °C and 10 000 x g. The supernatant was decanted, aspirated and the pellet was dried uncovered for 15 min at room temperature. The pellet was resuspended in 40 µl of TE (10 mM Tris, 2.5 mM EDTA).

For isolation of new sequences, the RNA integrity was checked on a 1.5 % agarose gel (1 x TAE, RNase free) that was run for 70 min at 50 V in an RNase free 1 x TAE buffer and stained with ethidiumbromide. The nucleic acid concentration was determined using a spectrophotometer (Nanodrop ND-1000, Isogen or DS-11, Denovix). gDNA contamination was removed using RQ1 RNase-Free DNase (Promega) according to the manufacturer's instructions. The absence of contaminating genomic DNA (gDNA) was confirmed by gel electrophoresis as described above. cDNA was synthesized using the iScript cDNA synthesis kit (Bio-rad) or using the AccuScript PfuUltra II RT-PCR Kit (Agilent), in case the cDNA was to be used for amplification of full length coding sequences (see further), according to the manufacturer's instructions.

For reverse transcription quantitative polymerase chain reaction (RT-qPCR) analysis, the nucleic acid concentration was determined using the DS-11 spectrophotometer (Denovix). gDNA was removed using the DNA-free kit (Ambion), according to the manufacturer’s *rigorous DNase treatment* instructions. The RNA concentration and quality were assessed using the Nanodrop spectrophotometer (Isogen) (Tables S1 and S2). The RNA integrity was assessed using the Experion microfluidic capillary electrophoresis system (Bio-Rad) in combination with the RNA StdSens Chips (Bio-Rad) (Figs. S1-S10). The samples were stored at -80 °C until cDNA synthesis.

First strand cDNA synthesis starting from 1000 ng of RNA was performed with the SuperScript III First-Strand Synthesis SuperMix (Invitrogen) according to the manufacturer’s instructions. Oligo(dT)20 was used for priming and all incubations occurred in a Perkin Elmer 2720 (Applied Biosystems). NoRTs of all samples were constructed using 1000 ng of RNA and nuclease-free water in a total volume of 20 µl.

## Isolation of new PIP coding sequences

The fragments listed in Table S3 were amplified, cloned into the pGEM®-T vector and Sanger sequenced by LGC Genomics, Berlin, Germany.

*Fragaria x ananassa* cv Diamante RNA was used for RACE PCR. The 5' RACE PCR and 3' RACE PCR protocols were adapted from ^1^ and ^2^ respectively. 20 to 60 times less RNA was used than described in these protocols, i.e. 75 to 250 ng of total RNA. In Table S4, the RACE primers are listed that were used in addition to the fixed primer and adaptor sequences.^1,2^ The 5' and 3' fragments were purified by adding 20 µl PCR product to 60 µl TE buffer (pH 8) and 40 µl poly (ethylene glycol) 8000 (30 %) 30 mM MgCl_2_ (Invitrogen), vortexing, centrifuging at 10 000 x g for 15 min and resuspending the pellet in 15 µl TE buffer. The purified PCR products were ligated into the pGEM®-T vector as described before and amplified in *E. coli* as described before. The plasmids were extracted as described before and the inserts were Sanger sequenced with the SP6 primer. For fragment D, 5’RACE was unsuccessful.

In case the RACE PCR resulted in sequences reaching beyond the start and stop codon, primers in the 5' and 3' untranslated region were designed for amplification of the full length coding sequence (Fragments A, B, C, E, Genbank Acc. No. GW403182.1; Table S5). In case the start- and/or stop codon was not found with RACE, primers were designed to amplify a partial coding sequence (Fragment D; Table S5). Amplification was done in *F. x ananassa* cv. Elsanta or Diamante (Table S5) using the AccuScript PfuUltra II RT-PCR Kit (Agilent), which contains a proofreading polymerase for both reverse transcription and amplification of cDNA fragments. PCR fragments were purified as described before, ligated into the pGEM®-T vector as described before and amplified in *E. coli* as described before. The inserts of two to nine positive colonies per PCR reaction, depending on the number of positive colonies available, were Sanger sequenced in both directions with primers SP6 and T7. The resulting sequences were named according to the current plant aquaporin nomenclature.^3^ Names were chosen to reflect the partitioning into different groups according to phylogenetic analysis and to reflect the similarities in coding sequence and untranslated regions (UTR). Very similar sequences were assigned the same name, followed by the suffix *–like* and a number.

## Plant material for RT-qPCR

Plant material was obtained from commercially grown greenhouse plants in Rumbeke (50°56’0”N, 3°9’13”E, Cléry) and Meigem (51°1'0"N, 3°30'28"E, Elsanta). The production system was soilless culture using peat as substrate, irrigation was automated and based on the irradiation sum (set point 1 MJ m^-^²), allowing a drainage percentage of 20-30 %. Fertigation was according to standard practices; the EC of the nutrient solution was 1.6 dS m^-1^.

At sampling time, mean stomatal conductance, measured for two mature and two young leaves per plant, was 147 ± 81 (SD) and 109 ± 45 (SD) mmol m^-2^ s^-1^) for Cléry and Elsanta respectively (AP4 Porometer, Delta T services, United Kingdom). Mean volumetric water content was 41 ± 4 vol% and 52 ± 11 (SD) vol% (HH2 Moisture Meter connected to a WET sensor, Delta T services, United Kingdom) for Cléry and Elsanta, respectively.

Roots (R) were washed under running tap water in order to remove most of the substrate and frozen in liquid nitrogen as soon as possible. They were sampled in three biological replicates (three separate plants). Cléry root samples were harvested on April 1^st^ 2015. Due to bad RNA quality (Figs S1–S4) Elsanta root samples from April 21^st^ were discarded and resampled on May 22^nd^ 2015, from different plants than those sampled on April 21^st^. The second sampling is referred to as biological replicates 4, 5 and 6.

## RT-qPCR

Nine candidate reference gene primer pairs were selected from literature. Primers were tested in an RT-qPCR assay on a subset of the 70-fold diluted samples (one sample per cultivar of each tissue) under the conditions described below. These data were used for reference gene selection using geNorm software. ^4^ GeNorm indicated that the use of two reference genes was sufficient. Based on this geNorm analyses, clathrin ((F) GAAGGTGTGCGCTCCCCATT; (R) CGCCAACCAACAGCACCTGT)^1^ and CHP3 ((F) TGTGGTCCAATGCCCATACTATT; (R) AACGGCTCCTCAGGAAGAGAA)^1,2^ were selected as reference genes (M-value = 0.463; CV-value = 0.161).

The amplification of all samples, noRTs and no template controls (NTC) was performed on a LightCycler 480 (Roche) in a white 384-well plate (Bio-rad) using the Maxima SYBR Green qPCR Master Mix (2X) (with separate ROX vial) (Life Technologies) according to the manufacturer's instructions in a 10 µl reaction volume. cDNA and noRT samples were diluted 70-fold. A noRT control was included for every gene/sample combination and two NTCs for each gene. Plates were sealed with an adhesive film. Cycling conditions were 10 min at 95 °C, followed by 50 cycles of 15 s at 95 °C, 30 s at 60 °C and 30 s at 72 °C. Data acquisition was done at the end of every cycle. Melting curve analysis was performed as follows: 5 s at 95 °C, 1 min at 65 °C and heating to 97 °C with a ramp rate of 0.06 °C s^-1^ to confirm a single specific product per primer pair. Data acquisition occurred 10 times for every °C. Data were analysed using the LightCycler480 software 1.62 (Roche). Samples were analysed in a single plate per gene. The 2^nd^ derivative maximization method was selected for Cq determination in every run.^5^ Samples were discarded in case the Cq-difference between sample and noRT was below 5 (ref. 6). When no amplification was detected, Cq values were manually set to 40.

Gene specific amplification efficiencies (E) (Table 1) and a normalization factor based on both validated reference genes was used for calculation of calibrated normalized relative quantities (CNRQ).^4^ Calibration was done to the minimum expression value of each gene. All calculations were done using qBASE+ software (Biogazelle).^6^ CNRQ-values were exported to Microsoft Excel. Biological replicates were averaged geometrically. Graphs were made using non-transformed CNRQ values in Sigmaplot 13.

Because of RNA degradation in the Elsanta root samples (biological replicates 4,5 and 6), all root samples were discarded and no root expression data was generated (Figs. S9-S10).

## Statistical analysis

First of all, for each aquaporin group/tissue combination, the expression in Elsanta was compared to the expression in Cléry. Next, for each aquaporin group/cultivar combination, several comparisons were made between tissues or groups of tissues. The expression in leaf tissue (Ly and Lm) was compared to the expression in the other tissues (P, sGF, lGF, WF, RF). The expression in young leaves (Ly) was compared to the expression in mature leaves (Lm). The expression in vegetative tissue (Ly, Lm, P) was compared to the expression in fruit tissue (sGF, lGF, WF, RF). Finally, pairwise comparisons of the four fruit developmental stages (sGF, lGF, WF and RF) were made.

## References

1 Scotto-Lavino E, Du G, Frohman M a. 5’ end cDNA amplification using classic RACE. *Nat Protoc* 2006; **1**: 2742–2745.

2 Scotto-Lavino E, Du G, Frohman M a. 3’ end cDNA amplification using classic RACE. *Nat Protoc* 2006; **1**: 2555–2562.

3 Johanson U, Karlsson M, Johansson I *et al.* The complete set of genes encoding major intrinsic proteins in Arabidopsis provides a framework for a new nomenclature for major intrinsic proteins in plants. *Plant Physiol* 2001; **126**: 1358–1369.

4 Vandesompele J, De Preter K, Pattyn F *et al.* Accurate normalization of real-time quantitative RT-PCR data by geometric averaging of multiple internal control genes. *Genome Biol* 2002; **3**: research0034.1–0034.11.

5 Luu-The V, Paquet N, Calvo E, Cumps J. Improved real-time RT-PCR method for high-throughput measurements using second derivative calculation and double correction. *Biotechniques* 2005; **38**: 287–293.

6 Hellemans J, Mortier G, De Paepe A, Speleman F, Vandesompele J. qBase relative quantification framework and software for management and automated analysis of real-time quantitative PCR data. *Genome Biol* 2007; **8**: R19.
