# Supplementary figures and images for: Identification and substrate prediction of new Fragaria x ananassa aquaporins and expression in different tissues and during strawberry fruit development
Source: Hortic Res. 2018 Apr 1;5:20. doi: 10.1038/s41438-018-0019-0 (PMC5880810; doi:10.1038/s41438-018-0019-0)

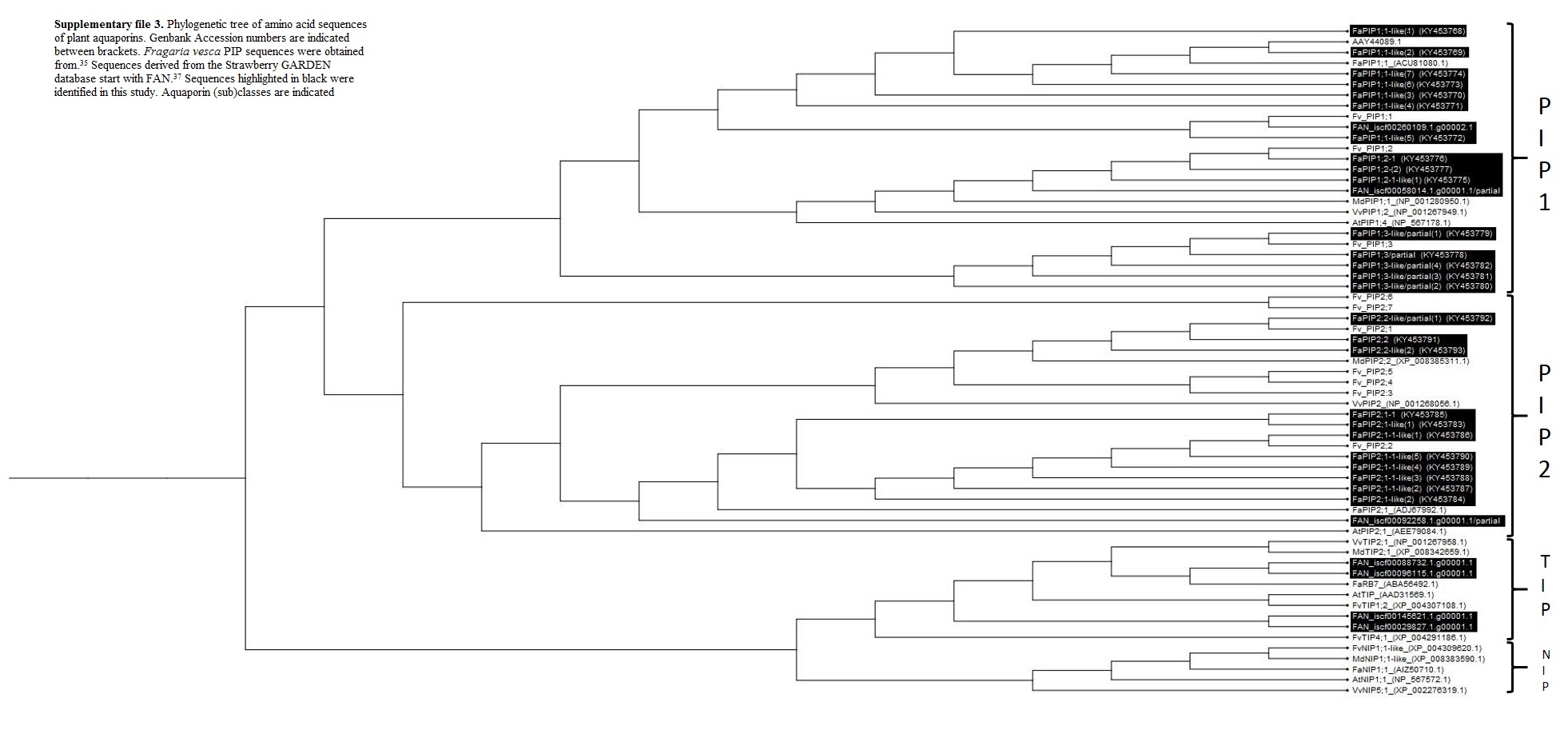

Supplement: Supplementary file 3 [file 41438_2018_19_MOESM3_ESM.jpg]
