# Supplementary material for: Identification and substrate prediction of new Fragaria x ananassa aquaporins and expression in different tissues and during strawberry fruit development
Source: Hortic Res. 2018 Apr 1;5:20. doi: 10.1038/s41438-018-0019-0 (PMC5880810; doi:10.1038/s41438-018-0019-0)

Article title: Identification and substrate prediction of new *Fragaria x ananassa* aquaporins and expression in different tissues and during strawberry fruit development

Journal: Horticulture Research

Authors: Britt Merlaen, Ellen De Keyser and Marie-Christine Van Labeke

Corresponding author: Marie-Christine Van Labeke, Plant Production, Faculty of Bioscience Engineering, Ghent University, Coupure Links 653, 9000 Gent, Belgium Email: mariechristine.vanlabeke@ugent.be

Supplementary file 4 Alignment of PIP cDNA sequences. Green boxes indicate Forward RT-qPCR primers as listed in Table 1. Red boxes indicate Reverse RT-qPCR primers as listed in Table 1.

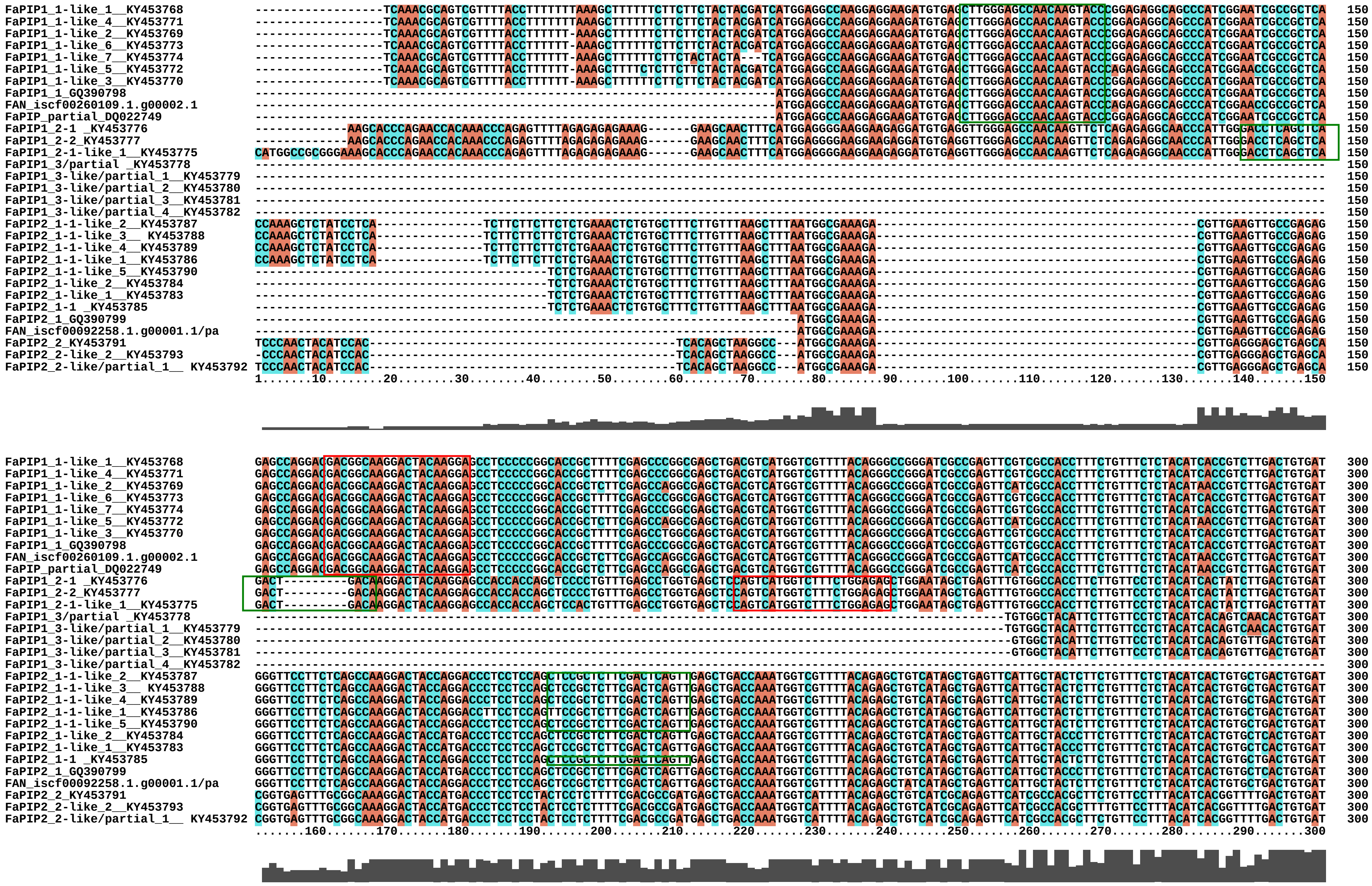
FaPIP1_1-like_1__KY453768
FaPIP1_1-like_4__KY453771
FaPIP1_1-like_2__KY453769
FaPIP1_1-like_6__KY453773
FaPIP1_1-like_7__KY453774
FaPIP1_1-like_5__KY453772
FaPIP1_1-like_3__KY453770
FaPIP1_1_GQ390798
FAN_iscf00260109.1.g00002.1
FaPIP_partial_DQ022749
FaPIP1_2-1 _KY453776
FaPIP1_2-2_KY453777
FaPIP1_2-1-like_1__KY453775
FaPIP1_3/partial _KY453778
FaPIP1_3-like/partial_1__KY453779
FaPIP1_3-like/partial_2__KY453780
FaPIP1_3-like/partial_3__KY453781
FaPIP1_3-like/partial_4__KY453782
FaPIP2_1-1-like_2__KY453787
FaPIP2_1-1-like_3__ KY453788
FaPIP2_1-1-like_4__KY453789
FaPIP2_1-1-like_1__KY453786
FaPIP2_1-1-like_5__KY453790
FaPIP2_1-like_2__KY453784
FaPIP2_1-like_1__KY453783
FaPIP2_1-1 _KY453785
FaPIP2_1_GQ390799
FAN_iscf00092258.1.g00001.1/pa
FaPIP2_2_KY453791
FaPIP2_2-like_2__KY453793
FaPIP2_2-like/partial_1__ KY453792
150
300

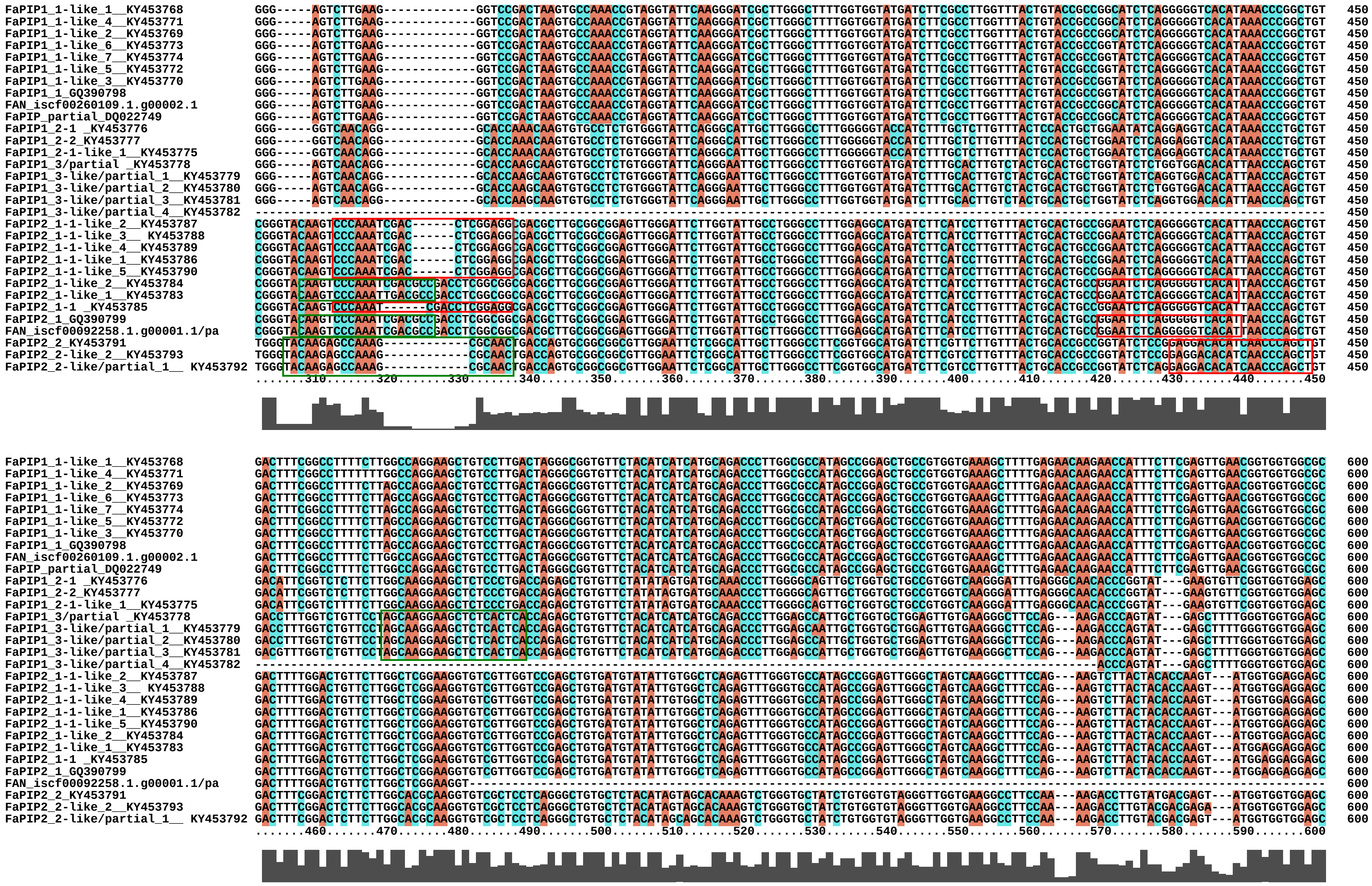
FaPIP1_1-like_1__KY453768
FaPIP1_1-like_4__KY453771
FaPIP1_1-like_2__KY453769
FaPIP1_1-like_6__KY453773
FaPIP1_1-like_7__KY453774
FaPIP1_1-like_5__KY453772
FaPIP1_1-like_3__KY453770
FaPIP1_1_GQ390798
FAN_iscf00260109.1.g00002.1
FaPIP_partial_DQ022749
FaPIP1_2-1 _KY453776
FaPIP1_2-2_KY453777
FaPIP1_2-1-like_1__KY453775
FaPIP1_3/partial _KY453778
FaPIP1_3-like/partial_1__KY453779
FaPIP1_3-like/partial_2__KY453780
FaPIP1_3-like/partial_3__KY453781
FaPIP1_3-like/partial_4__KY453782
FaPIP2_1-1-like_2__KY453787
FaPIP2_1-1-like_3__ KY453788
FaPIP2_1-1-like_4__KY453789
FaPIP2_1-1-like_1__KY453786
FaPIP2_1-1-like_5__KY453790
FaPIP2_1-like_2__KY453784
FaPIP2_1-like_1__KY453783
FaPIP2_1-1 _KY453785
FaPIP2_1_GQ390799
FAN_iscf00092258.1.g00001.1/pa
FaPIP2_2_KY453791
FaPIP2_2-like_2__KY453793
FaPIP2_2-like/partial_1__ KY453792
450
600

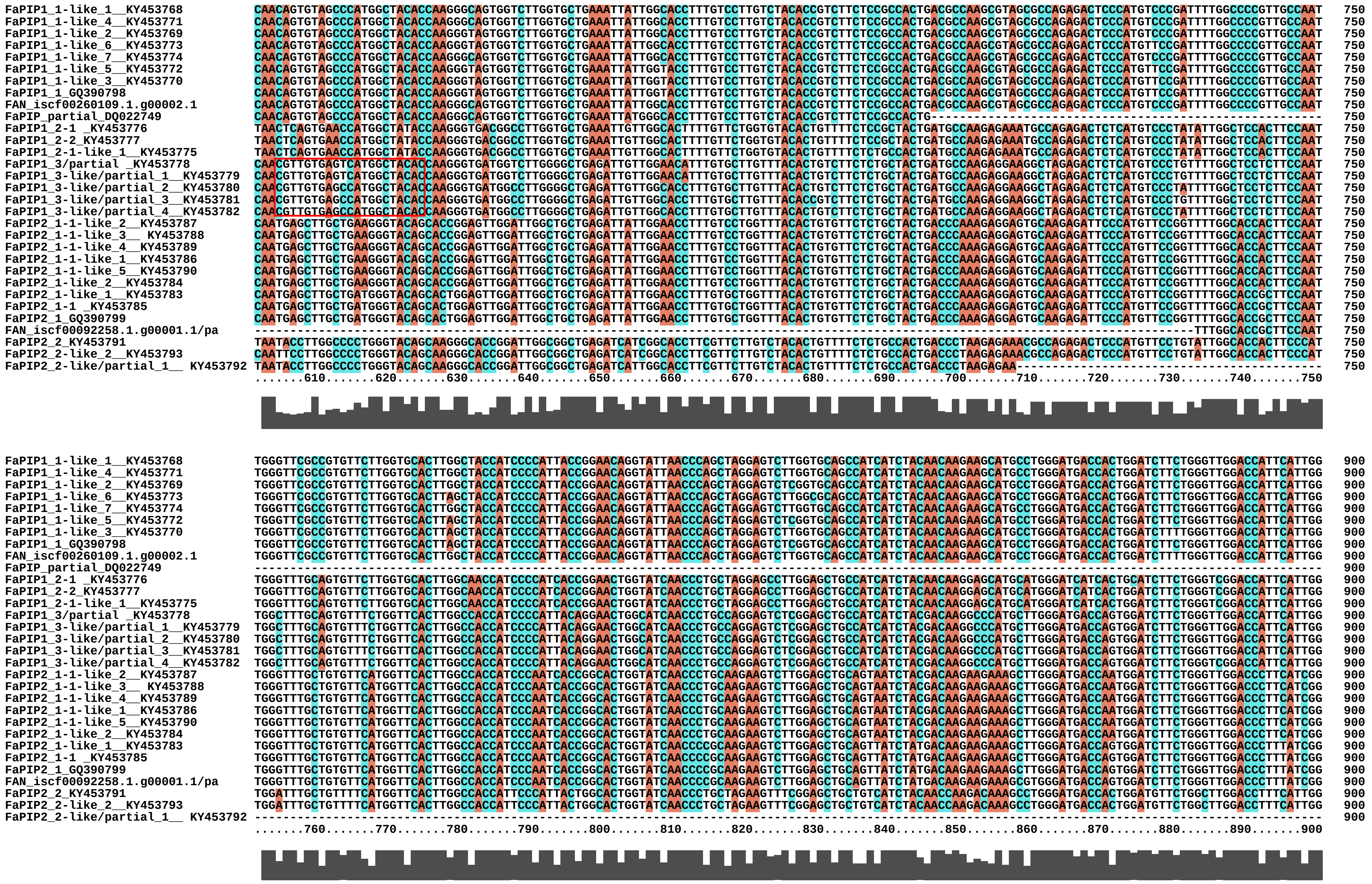
FaPIP1_1-like_1__KY453768
FaPIP1_1-like_4__KY453771
FaPIP1_1-like_2__KY453769
FaPIP1_1-like_6__KY453773
FaPIP1_1-like_7__KY453774
FaPIP1_1-like_5__KY453772
FaPIP1_1-like_3__KY453770
FaPIP1_1_GQ390798
FAN_iscf00260109.1.g00002.1
FaPIP_partial_DQ022749
FaPIP1_2-1 _KY453776
FaPIP1_2-2_KY453777
FaPIP1_2-1-like_1__KY453775
FaPIP1_3/partial _KY453778
FaPIP1_3-like/partial_1__KY453779
FaPIP1_3-like/partial_2__KY453780
FaPIP1_3-like/partial_3__KY453781
FaPIP1_3-like/partial_4__KY453782
FaPIP2_1-1-like_2__KY453787
FaPIP2_1-1-like_3__ KY453788
FaPIP2_1-1-like_4__KY453789
FaPIP2_1-1-like_1__KY453786
FaPIP2_1-1-like_5__KY453790
FaPIP2_1-like_2__KY453784
FaPIP2_1-like_1__KY453783
FaPIP2_1-1 _KY453785
FaPIP2_1_GQ390799
FAN_iscf00092258.1.g00001.1/pa
FaPIP2_2_KY453791
FaPIP2_2-like_2__KY453793
FaPIP2_2-like/partial_1__ KY453792
750
610 620 630 640 650 660 670 680 690 700 710 720 730 740 750
900
760 770 780 790 800 810 820 830 840 850 860 870 880 890 900

```
FaPIP1_1-like_1__KY453768          TGCAGCACTTGCAGCTTTGTACCACGTCGTCGTGATCAGGGCCATTCCTTTCAAGAATAAG---TGAATGCT-----------------------------------------------TAAGCAGT-----------------------  1050
FaPIP1_1-like_4__KY453771          TGCAGCACTTGCAGCTTTGTACCACGTCGTCGTGATCAGGGCCATTCCTTTCAAGAATAAG---TGAATGCT-----------------------------------------------TAAGCAGT-----------------------  1050
FaPIP1_1-like_2__KY453769          TGCAGCACTTGCAGCTTTGTACCACGTCGTCGTGATCAGGGCCATTCCTTTCAAGAATAAG---TGAATGCT-----------------------------------------------TAAGCAGT-----------------------  1050
FaPIP1_1-like_6__KY453773          TGCAGCACTTGCAGCTTTGTACCACGTCGTCGTGATCAGGGCCATTCCTTTCAAGAATAAG---TGAATGCT-----------------------------------------------TAAGCAGT-----------------------  1050
FaPIP1_1-like_7__KY453774          TGCAGCACTTGCAGCTTTGTACCATGTGGTTGTGATCAGGGCCATTCCCTTCAAGAACAAG---TGAATGCT-----------------------------------------------TAAGCAGT-----------------------  1050
FaPIP1_1-like_5__KY453772          TGCAGCACTTGCAGCTTTGTACCACGTCGTCGTGATCAGGGCCATTCCTTTCAAGAATAAG---TGAATGCT-----------------------------------------------TAAGCAGT-----------------------  1050
FaPIP1_1-like_3__KY453770          TGCAGCACTTGCAGCTCTGTACCATGTCGTCGTGATCAGGGCCATTCCTTTCAAGAATAAG---TGAATGCT-----------------------------------------------TAAGCATT-----------------------  1050
FaPIP1_1_GQ390798                  TGCAGCACTTGCAGCTTTGTACCACGTCGTCGTGATCAGGGCCATTCCTTTCAAGAATAAG---TGA---------------------------------------------------------------------------------  1050
FAN_iscf00260109.1.g00002.1        TGCAGCACTTGCGGCTCTGTACCACGTCGTCGTGATCAGGGCCATTCCTTTCAAGAATAAG---TGA---------------------------------------------------------------------------------  1050
FaPIP_partial_DQ022749             ------------------------------------------------------------------------------------------------------------------------------------------------------  1050
FaPIP1_2-1 _KY453776               AGCTGCACTTGCTGCCCTCTACCATCAGATAGTCATCAGAGCCATTCCATTCAAGACCAGAGATTGAGAGAT---CCTATTAGTCTCTCCTCGTTTAGACTTGCATCTAGGCTGGATTATATCTATT-----------------------  1050
FaPIP1_2-2_KY453777                AGCTGCACTTGCTGCCCTCTACCATCAGATAGTCATCAGAGCCATTCCATTCAAGACCAGGGATTGAGAGATGCACTTGCTGCCCTCTCCTCGTTTACACTTGC--------TGGATTATATCTATT-----------------------  1050
FaPIP1_2-1-like_1__KY453775        AGCTGCACTTGCTGCCCTCTACCATCAGATAGTCATCAGAGCCATTCCATTCAAGACCAGGGATTGAGAGAT---CCTATAAGTCTCTCCTCATTTAGACTTGCTTCTAGGCTGGATTATATATATT-----------------------  1050
FaPIP1_3/partial _KY453778         TGCTGCACTTGCTGCTTTCTACCACATGGTAGTCATCAGGGCCATA--------------------------------------------------------------------------------------------------------  1050
FaPIP1_3-like/partial_1__KY453779  TGCTGCACTTGCTGCTTTCTACCACATGGTAGTCATCAGGGCCATT--------------------------------------------------------------------------------------------------------  1050
FaPIP1_3-like/partial_2__KY453780  TGCTGCACTTGCTGCTTTCTACCACACGGTAGTCATCAGGGCCAT---------------------------------------------------------------------------------------------------------  1050
FaPIP1_3-like/partial_3__KY453781  TGCTGCACTTGCTGCTTTCTACCACATGGTAGTCATCAGGGCCATT--------------------------------------------------------------------------------------------------------  1050
FaPIP1_3-like/partial_4__KY453782  TGCTGCACTTGCTGCTTTCTACCACATGGTAGTCATCAGGGCCA----------------------------------------------------------------------------------------------------------  1050
FaPIP2_1-1-like_2__KY453787        TGCCGCAATTGCAGCCTTCTACCACCAATTCATCTTGAGAGCAGGAGCTGTTAAAG-------------------------------CGCTCGGGTCATTCAGGAGCAACCCCCATAATTGAACATTTTCAGAGTCACAATGAAAAGATG  1050
FaPIP2_1-1-like_3__ KY453788       TGCCGCAATTGCAGCCTTCTACCACCAATTCATCTTGAGAGCAGGAGCTGTTAAAG-------------------------------CGCTCGGGTCATTCAGGAGCAACCCCCATAATTGAACATTTTCAGAGTCACAATGAAA-GATG  1050
FaPIP2_1-1-like_4__KY453789        TGCCGCAATTGCAGCCTTCTACCACCAATTCATCTTGAGAGCAGGAGCTGTTAAAG-------------------------------CGCTCGGGTCATTCAGGAGCAACCCCCATAATTGAACATTTTCAGAGTCACAATGAAA-GATG  1050
FaPIP2_1-1-like_1__KY453786        TGCCGCAATTGCAGCCTTCTACCACCAATTCATCTTGAGAGCAGGAGCTGTTAAAG-------------------------------CGCTCGGGTCATTCAGGAGCAACCCCCATAATTGAACATTTTCAGAGTCACAATGAAA-GATG  1050
FaPIP2_1-1-like_5__KY453790        TGCCGCAATTGCAGCCTTCTACCACCAATTCATCTTGAGAGCAGGAGCTGTTAAAG-------------------------------CGCTCGGGTCATTCAGGAGCAACCCCCATAATTGAACATTTTCAGAGTCACAATGAAA-GATG  1050
FaPIP2_1-like_2__KY453784          TGCCGCAATTGCAGCCTTCTACCACCAATTCATCTTGAGAGCAGGAGCTGTTAAAG-------------------------------CGCTCGGGTCATTCAGGAGCAACCCCCATAATTGAACATTTTCAGAGTCACAATGAAA-GATG  1050
FaPIP2_1-like_1__KY453783          TGCAGCAATTGCAGCCTTCTACCACCAATTCATCTTGAGAGCAGGAGCTGTTAAAG-------------------------------CGCTCGGGTCATTCAGGAGCAACCCTCATAATTGAACATTTTCAGAGTCACAATGAAA-GATG  1050
FaPIP2_1-1 _KY453785               TGCAGCAATTGCAGCCTTCTACCACCAATTCATCTTGAGAGCAGGAGCTGTTAAAG-------------------------------CGCTCGGGTCATTCAGGAGCAACCCCCATAATTGAACATTTTCAGAGTCACAATGAAA-GATG  1050
FaPIP2_1_GQ390799                  TGCAGCAATTGCAGCCTTCTACCACCAATTCATCTTGAGAGCAGGAGCTGTTAAAG-------------------------------CGCTCGGGTCTTTCAGGAGCAATGCTTA-----------------------------------  1050
FAN_iscf00092258.1.g00001.1/pa     TGCAGCAATTGCAGCCTTCTACCACCAATTCATCTTGAGAGCAGGAGCTGTTAAAG-------------------------------CGCTCGG--------------------------------------------------------  1050
FaPIP2_2_KY453791                  AGCTGCCATTGCTGCCTTTTACCACCAATTTGTTCTGAGGGCAGGAGCAATCAAGG-------------------------------CCCTAGGGTCTTTCAGGAGCAATGCTTAA----------------------------------  1050
FaPIP2_2-like_2__KY453793          AGCTGCCATTGCTGCCTTTTACCACCAATTTGTTCTGAGGGCAGGAGCAATCAAGG-------------------------------CCCTAGGGTCTTTCAGGAGCAATGCTTAA----------------------------------  1050
FaPIP2_2-like/partial_1__ KY453792 ------------------------------------------------------------------------------------------------------------------------------------------------------  1050
                                   .......910.......920.......930.......940.......950.......960.......970.......980.......990......1000......1010......1020......1030......1040......1050
```

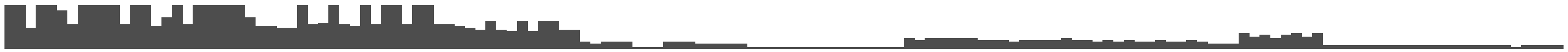

```
FaPIP1_1-like_1__KY453768          ------------------------------------------------------------------------------------------------------------------------------------------------------  1200
FaPIP1_1-like_4__KY453771          ------------------------------------------------------------------------------------------------------------------------------------------------------  1200
FaPIP1_1-like_2__KY453769          ------------------------------------------------------------------------------------------------------------------------------------------------------  1200
FaPIP1_1-like_6__KY453773          ------------------------------------------------------------------------------------------------------------------------------------------------------  1200
FaPIP1_1-like_7__KY453774          ------------------------------------------------------------------------------------------------------------------------------------------------------  1200
FaPIP1_1-like_5__KY453772          ------------------------------------------------------------------------------------------------------------------------------------------------------  1200
FaPIP1_1-like_3__KY453770          ------------------------------------------------------------------------------------------------------------------------------------------------------  1200
FaPIP1_1_GQ390798                  ------------------------------------------------------------------------------------------------------------------------------------------------------  1200
FAN_iscf00260109.1.g00002.1        ------------------------------------------------------------------------------------------------------------------------------------------------------  1200
FaPIP_partial_DQ022749             ------------------------------------------------------------------------------------------------------------------------------------------------------  1200
FaPIP1_2-1 _KY453776               ------------------------------------------------------------------------------------------------------------------------------------------------------  1200
FaPIP1_2-2_KY453777                ------------------------------------------------------------------------------------------------------------------------------------------------------  1200
FaPIP1_2-1-like_1__KY453775        ------------------------------------------------------------------------------------------------------------------------------------------------------  1200
FaPIP1_3/partial _KY453778         ------------------------------------------------------------------------------------------------------------------------------------------------------  1200
FaPIP1_3-like/partial_1__KY453779  ------------------------------------------------------------------------------------------------------------------------------------------------------  1200
FaPIP1_3-like/partial_2__KY453780  ------------------------------------------------------------------------------------------------------------------------------------------------------  1200
FaPIP1_3-like/partial_3__KY453781  ------------------------------------------------------------------------------------------------------------------------------------------------------  1200
FaPIP1_3-like/partial_4__KY453782  ------------------------------------------------------------------------------------------------------------------------------------------------------  1200
FaPIP2_1-1-like_2__KY453787        TTGCTTCCACTTCATCATGGTTTCAGGCCTTTCAGGTTGGGAAACAATGAAGAGTCTATGCACTTTGGAAAGAATAACGGGGTAGTACTTGTGGTAGCTAGCTTATTATGCTTGTGTATGGAGGTTAAATTTCTTTTCCTGGTATCTCAA  1200
FaPIP2_1-1-like_3__ KY453788       TTGCTTCCACTTCATCATGGCTTCAGGCCTTTCAGGTTGGGAAACAATGAAGAGTCTATGCACTTTGGAAAGAATAACGGGGTAGTACTTGTGGTAGCTAGCTTATTATGCTTGTGTATGGAGGTTAAATTTCTTTTCCTGGTATCTCAA  1200
FaPIP2_1-1-like_4__KY453789        TTGCTTCCACTTCATCATGGTTTCAGGCCTTTCAGGTTGGGAAACAATGAAGAGTCTATGCACTTTGGAAAGAATAACGGGGTAGTACTTGTGGTAGCTAGCTTATTATGCTTGTGTATGGAGGTTAAATTTCTTTTCCTGGTATCTCAA  1200
FaPIP2_1-1-like_1__KY453786        TTGCTTCCACTTCATCATGGTTTCAGGCCTTTCAGGTTGGGAAACAATGAAGAGTCTATGCACTTTGGAAAGAATAACGGGGTAGTACTTGTGGTAGCTAGCTTATTATGCTTGTGTATGGAGGTTAAATTTCTTTTCCTGGTATCTCAA  1200
FaPIP2_1-1-like_5__KY453790        TTGCTTCCAC--------------------------------------------------------------------------------------------------------------------------------------------  1200
FaPIP2_1-like_2__KY453784          TTGCTTCCAC--------------------------------------------------------------------------------------------------------------------------------------------  1200
FaPIP2_1-like_1__KY453783          TTGCTTCCAC--------------------------------------------------------------------------------------------------------------------------------------------  1200
FaPIP2_1-1 _KY453785               TTGCTTCCAC--------------------------------------------------------------------------------------------------------------------------------------------  1200
FaPIP2_1_GQ390799                  ------------------------------------------------------------------------------------------------------------------------------------------------------  1200
FAN_iscf00092258.1.g00001.1/pa     ------------------------------------------------------------------------------------------------------------------------------------------------------  1200
FaPIP2_2_KY453791                  ----TTTCACTTAAGCAGGATTTTCCACCCA-----------------------------------------------------------------------------------------------------------------------  1200
FaPIP2_2-like_2__KY453793          ----TTTCACTTAAGCAGGATTTTCCACCCA-----------------------------------------------------------------------------------------------------------------------  1200
FaPIP2_2-like/partial_1__ KY453792 ------------------------------------------------------------------------------------------------------------------------------------------------------  1200
                                   ......1060......1070......1080......1090......1100......1110......1120......1130......1140......1150......1160......1170......1180......1190......1200
```

```
FaPIP1_1-like_1__KY453768          ----GAGTGATGGTTTCAACAATCAAC---------AGCTT-  1242
FaPIP1_1-like_4__KY453771          ----GAGTGATGGTTTCAACAATCAAC---------AGCTT-  1242
FaPIP1_1-like_2__KY453769          ----GAGTGATGGTTTCAACAATCAAC---------AGCTT-  1242
FaPIP1_1-like_6__KY453773          ----GAGTGATGGTTTCAACAATCAAC---------AGCTTT  1242
FaPIP1_1-like_7__KY453774          ----GAGTGAGCGTTTCAACAATCAAC---------AGCTTT  1242
FaPIP1_1-like_5__KY453772          ----GAGTGATGGTTTCAACAATCAAC---------AGCTTT  1242
FaPIP1_1-like_3__KY453770          ----GAGTGAGGGTTTCAACAATCAAC---------AGCTT-  1242
FaPIP1_1_GQ390798                  ------------------------------------------  1242
FAN_iscf00260109.1.g00002.1        ------------------------------------------  1242
FaPIP_partial_DQ022749             ------------------------------------------  1242
FaPIP1_2-1 _KY453776               ----TTGTTATGTATTCATGTTGTGACTGGGATTGAAGAGTT  1242
FaPIP1_2-2_KY453777                ----TTGTTATGTATTCATGTTGTGACTGGGATTGAAGAGTT  1242
FaPIP1_2-1-like_1__KY453775        ----TTGTTATGTATTCATGTTGTGACTGGGATTGAAGAGTT  1242
FaPIP1_3/partial _KY453778         ------------------------------------------  1242
FaPIP1_3-like/partial_1__KY453779  ------------------------------------------  1242
FaPIP1_3-like/partial_2__KY453780  ------------------------------------------  1242
FaPIP1_3-like/partial_3__KY453781  ------------------------------------------  1242
FaPIP1_3-like/partial_4__KY453782  ------------------------------------------  1242
FaPIP2_1-1-like_2__KY453787        TTTGGTCCAGTAGAGGGATTGAGTGAGCGTTTCAACAATCC-  1242
FaPIP2_1-1-like_3__ KY453788       TTTGGTCCAGTAGAGGGATTGAGTGAGCGTTTCAACAATCAA  1242
FaPIP2_1-1-like_4__KY453789        TTTGGTCCAGTAGAGGGATTGAGTGAGCGTTTCAACAATCA-  1242
FaPIP2_1-1-like_1__KY453786        TTTGGTCCAGTAGAGGGATTGAGTGAGCGTTTCAACAATCAA  1242
FaPIP2_1-1-like_5__KY453790        -----------------------------------------A  1242
FaPIP2_1-like_2__KY453784          ------------------------------------------  1242
FaPIP2_1-like_1__KY453783          ------------------------------------------  1242
FaPIP2_1-1 _KY453785               ------------------------------------------  1242
FaPIP2_1_GQ390799                  -----------------------------------------A  1242
FAN_iscf00092258.1.g00001.1/pa     ------------------------------------------  1242
FaPIP2_2_KY453791                  ------------------------------------AACTTG  1242
FaPIP2_2-like_2__KY453793          ------------------------------------AACTTG  1242
FaPIP2_2-like/partial_1__ KY453792 ------------------------------------------  1242
                                   ......1210......1220......1230......1240..
```

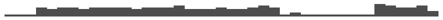

Supplement: Supplementary file 4 [file 41438_2018_19_MOESM4_ESM.pdf]
