# Supplementary material for: Identification and substrate prediction of new Fragaria x ananassa aquaporins and expression in different tissues and during strawberry fruit development
Source: Hortic Res. 2018 Apr 1;5:20. doi: 10.1038/s41438-018-0019-0 (PMC5880810; doi:10.1038/s41438-018-0019-0)
Supplement: Supplementary file 5 — Supplementary figures [file 41438_2018_19_MOESM5_ESM.docx]

**Article title**: Identification and substrate prediction of new *Fragaria x ananassa* aquaporins and expression in different tissues and during strawberry fruit development

**Journal**: Horticulture Research

**Authors**: Britt Merlaen, Ellen De Keyser and Marie-Christine Van Labeke

**Corresponding author**: Marie-Christine Van Labeke, Plant Production, Faculty of Bioscience Engineering, Ghent University, Coupure Links 653, 9000 Gent, Belgium Email: mariechristine.vanlabeke@ugent.be

**Supplementary figures** Figs. S1 - S10


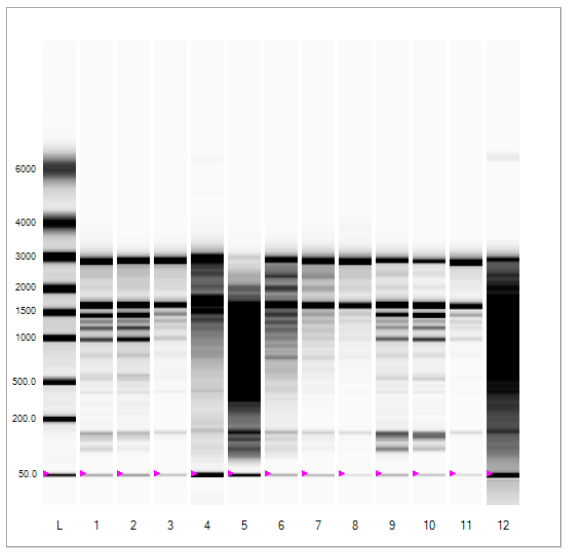


R (2)

P (2)

Lm (2)

Ly (2)

RF (1)

WF (1)

lGF (1)

Lm (1)

R (1)

sGF (1)

Ly (1)

P (1)

**Fig. S 1** **Virtual gel image of Elsanta DNase treated RNA samples (part 1).** Ly = young leaf; Lm = mature leaf; P = petiole; R= root; sGF = small green fruit; lGF = large green fruit; WF = white fruit; RF = red fruit. The numbers between brackets indicate the biological replicate. Estimated sizes of marker bands (lane L) are presented in kilodaltons (kDa).


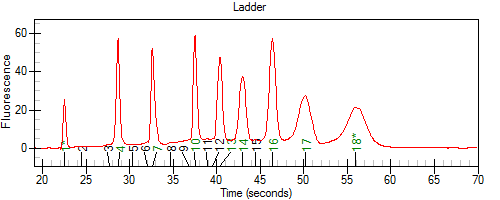

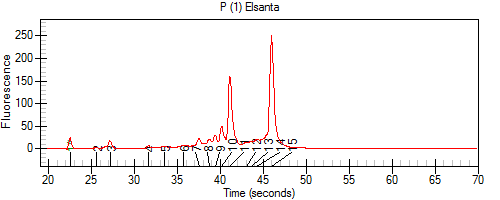

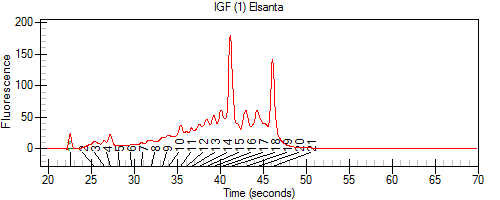

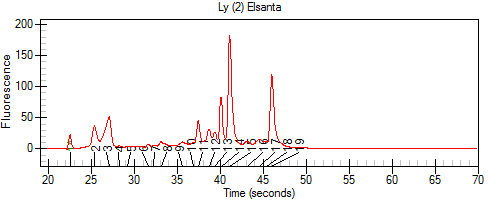

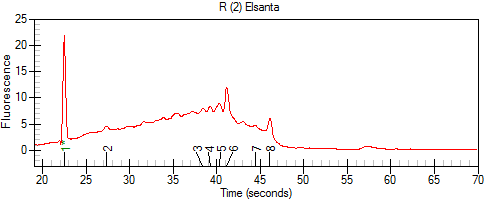

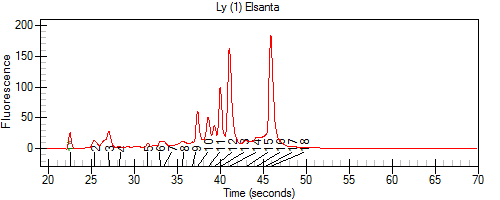

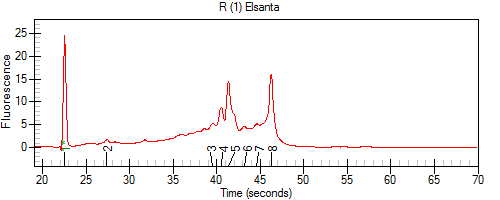

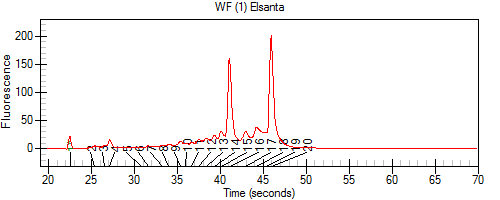

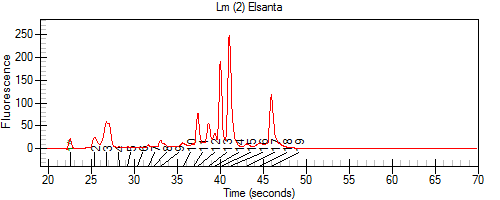

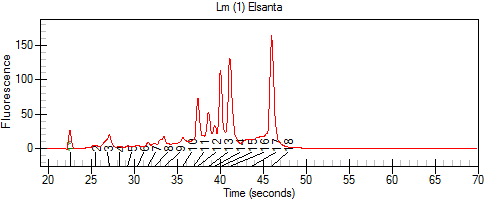

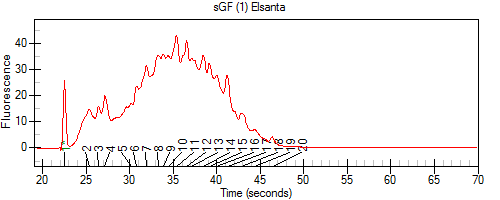

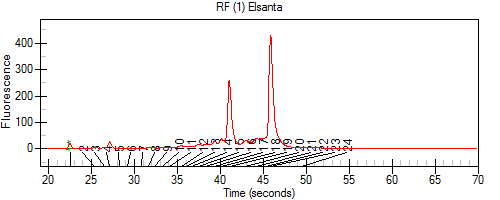

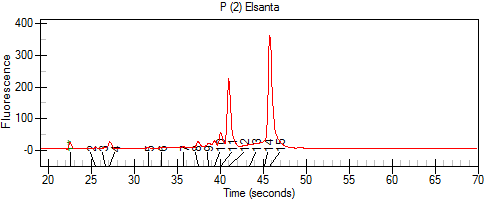


**Fig. S 2** **Electropherograms of Elsanta DNase treated RNA samples (part 1).** Ly = young leaf; Lm = mature leaf; P = petiole; R= root; sGF = small green fruit; lGF = large green fruit; WF = white fruit; RF = red fruit. The numbers between brackets indicate the biological replicate.

WF (2)

RF (3)

WF (3)

sGF (3)

lGF (3)

R (3)

P (3)

Lm (3)

Ly (3)

RF(2)

lGF (2)

sGF (2)

**Fig. S 3** **Virtual gel image of Elsanta DNase treated RNA samples (part 2).** Ly = young leaf; Lm = mature leaf; P = petiole; R= root; sGF = small green fruit; lGF = large green fruit; WF = white fruit; RF = red fruit. The numbers between brackets indicate the biological replicate. Estimated sizes of marker bands (lane L) are presented in kilodaltons (kDa).


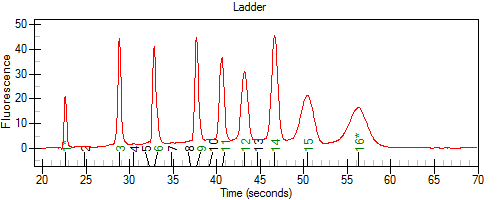

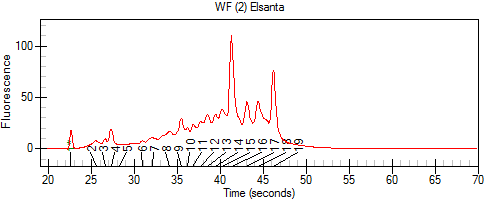

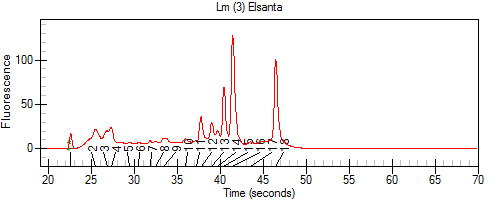

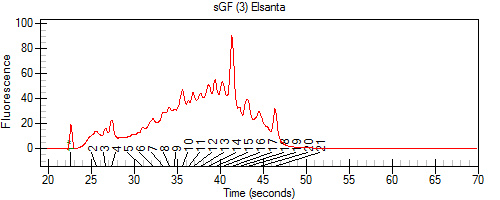

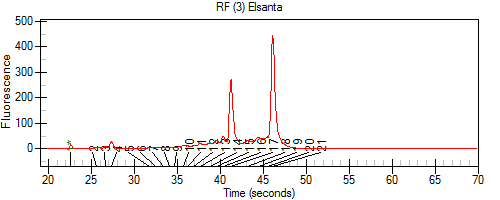

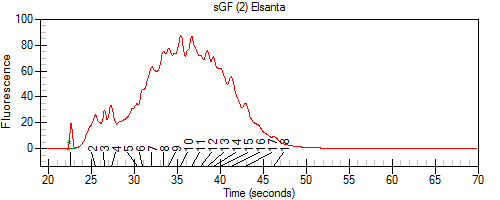

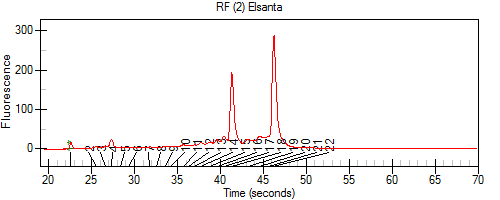

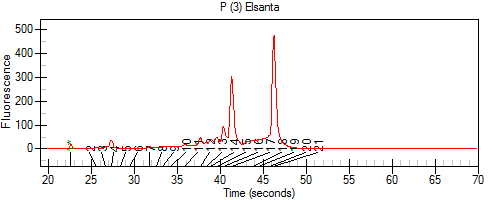

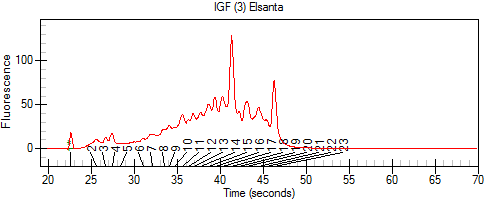

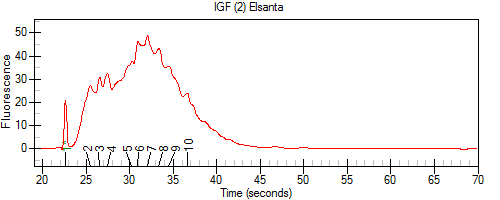

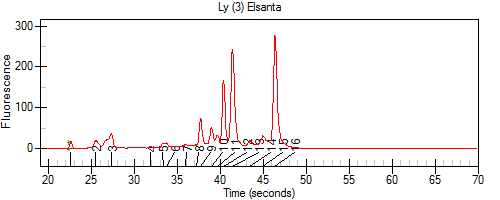

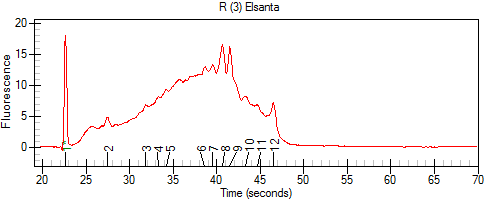

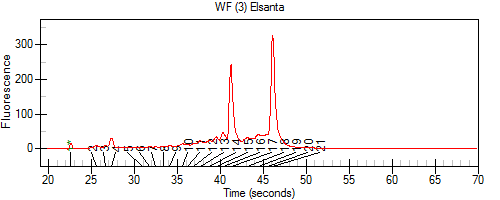


**Fig. S 4** **Electropherograms of Elsanta DNase treated RNA samples (part 2).** Ly = young leaf; Lm = mature leaf; P = petiole; R= root; sGF = small green fruit; lGF = large green fruit; WF = white fruit; RF = red fruit. The numbers between brackets indicate the biological replicate.

Ly (1)

P (1)

R (1)

sGF (1)

lGF (1)

WF (1)

RF (1)

P (2)

R (2)

Lm (1)

**Fig. S 5** **Virtual gel image of Cléry DNase treated RNA samples (part 1).** Ly = young leaf; Lm = mature leaf; P = petiole; R= root; sGF = small green fruit; lGF = large green fruit; WF = white fruit; RF = red fruit. The numbers between brackets indicate the biological replicate. Estimated sizes of marker bands (lane L) are presented in kilodaltons (kDa). Lanes 9 and 10 had technical issues.


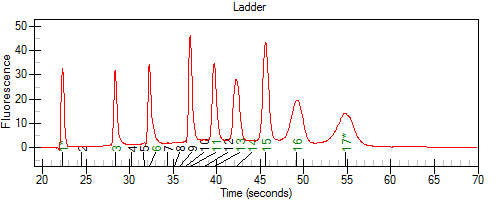

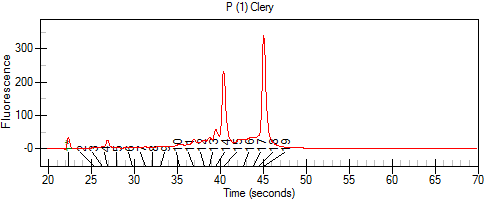

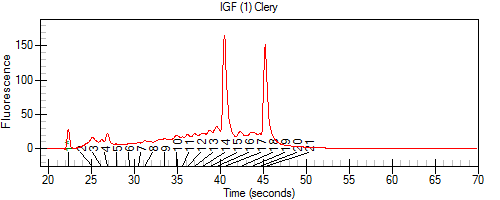

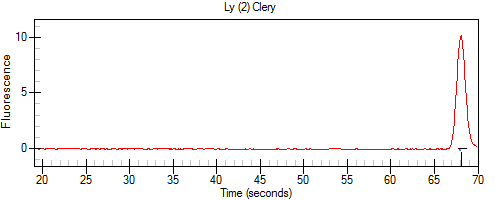

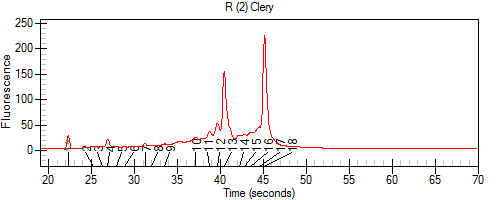

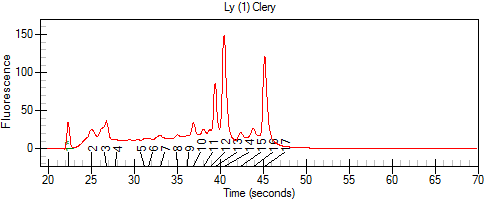

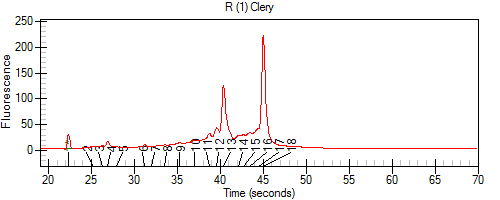

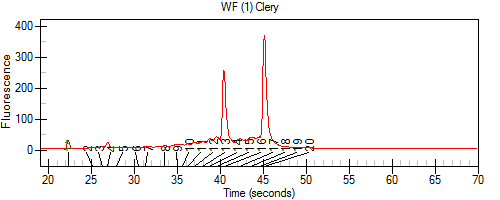

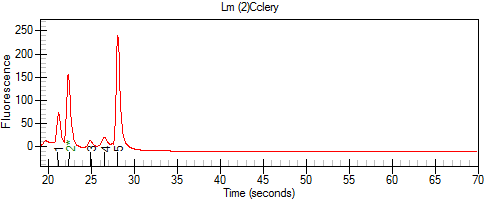

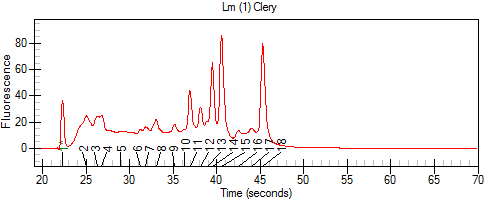

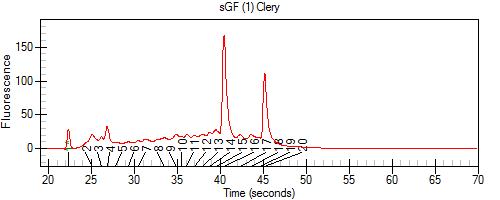

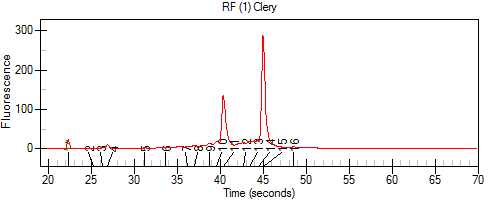

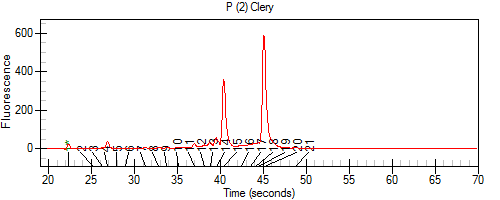


**Fig. S 6** **Electropherograms of Cléry DNase treated RNA samples (part 1).** Ly = young leaf; Lm = mature leaf; P = petiole; R= root; sGF = small green fruit; lGF = large green fruit; WF = white fruit; RF = red fruit. The numbers between brackets indicate the biological replicate. Ly(2) Cléry and Lm (2) Cléry had technical issues.

WF (2)

RF (3)

WF (3)

sGF (3)

lGF (3)

R (3)

P (3)

Lm (3)

Ly (3)

RF(2)

lGF (2)

sGF (2)

**Fig. S 7** **Virtual gel image of Cléry DNase treated RNA samples (part 2).** Ly = young leaf; Lm = mature leaf; P = petiole; R= root; sGF = small green fruit; lGF = large green fruit; WF = white fruit; RF = red fruit. The numbers between brackets indicate the biological replicate. Estimated sizes of marker bands (lane L) are presented in kilodaltons (kDa).


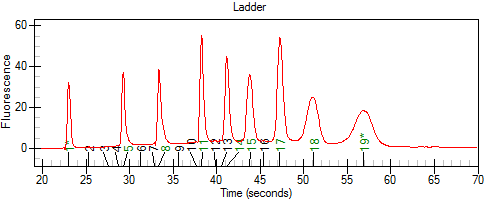

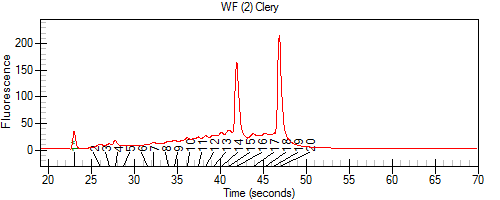

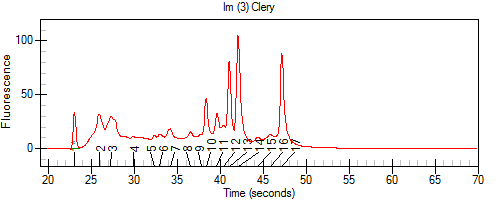

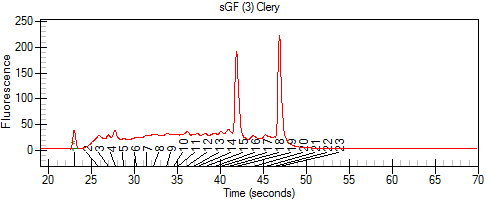

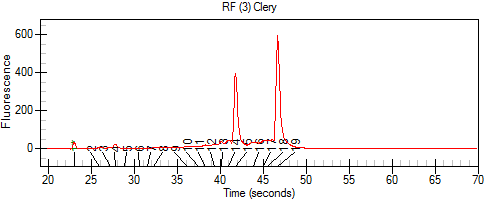

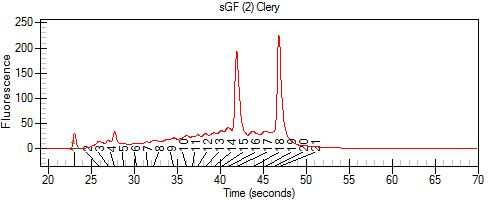

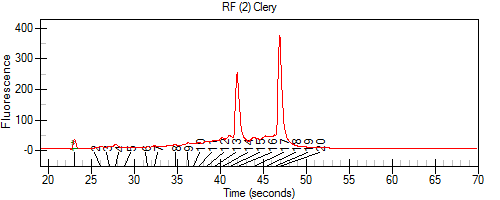

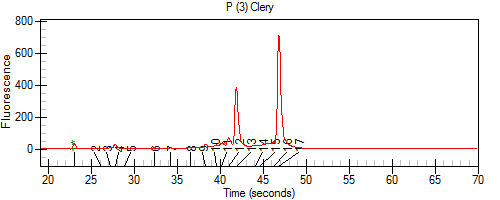

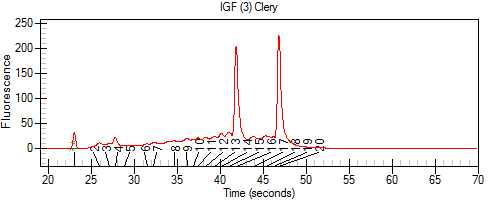

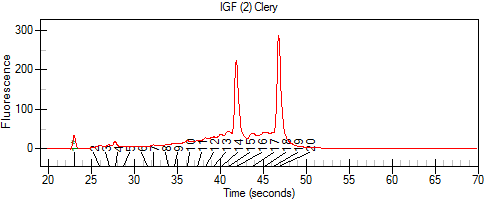

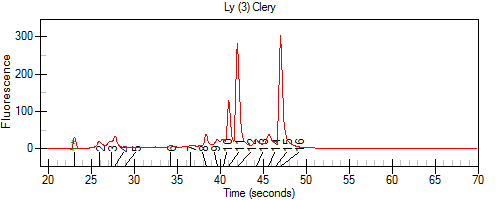

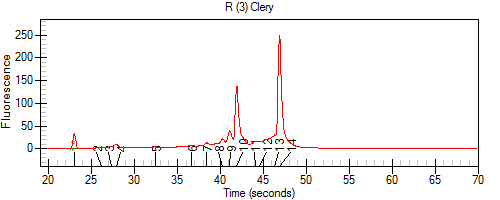

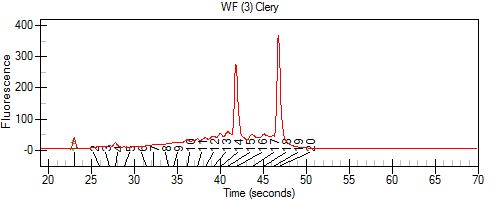


**Fig. S 8** **Electropherograms of Cléry DNase treated RNA samples (part 2).** Ly = young leaf; Lm = mature leaf; P = petiole; R= root; sGF = small green fruit; lGF = large green fruit; WF = white fruit; RF = red fruit. The numbers between brackets indicate the biological replicate.

Cléry

Elsanta

R (6)

sGF (4)

lGF (6)

Lm (2)

R (5)

R (4)

sGF (6)

lGF (4)

Ly (2)

sGF (5)

lGF (5)

**Fig. S 9** **Virtual gel image of Elsanta and Cléry DNase treated RNA samples.** Ly = young leaf; Lm = mature leaf; P = petiole; R= root; sGF = small green fruit; lGF = large green fruit; WF = white fruit; RF = red fruit. The numbers between brackets indicate the biological replicate. Estimated sizes of marker bands (lane L) are presented in kilodaltons (kDa).


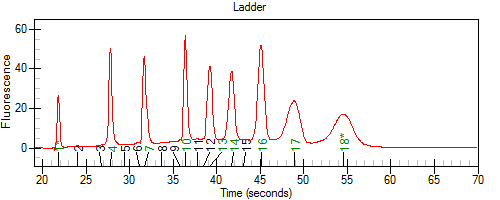

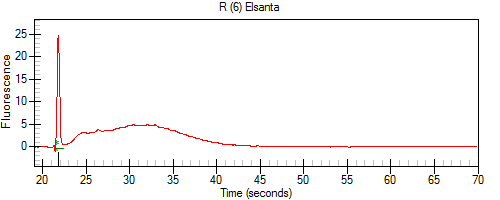

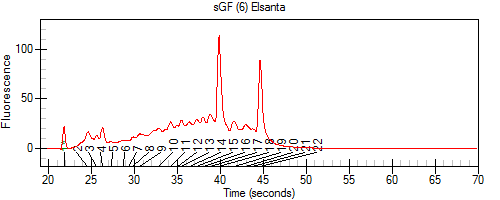

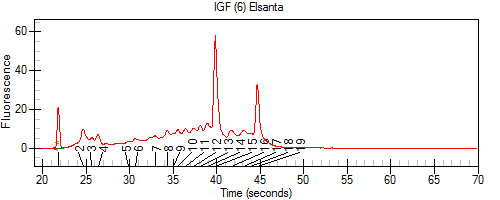

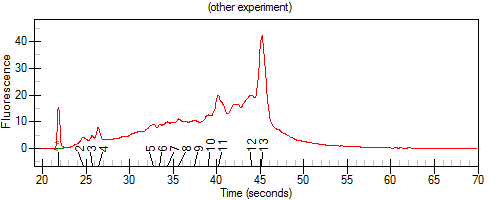

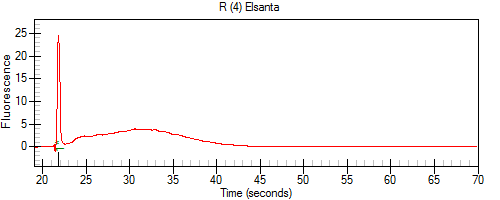

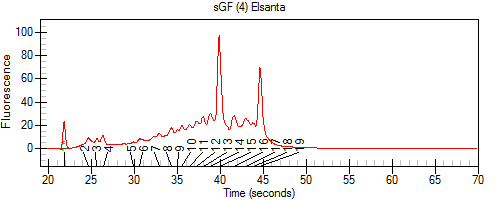

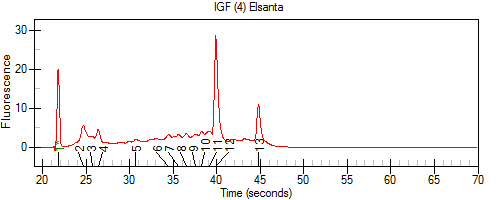

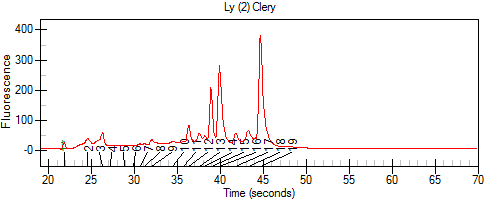

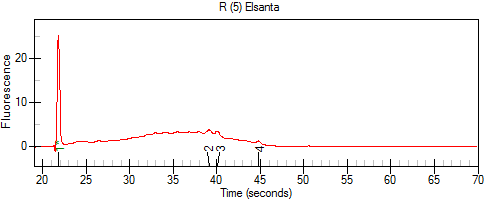

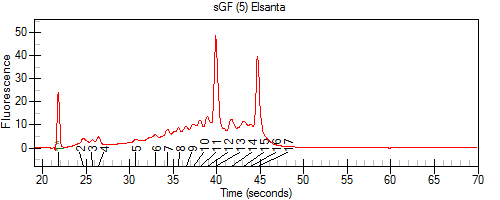

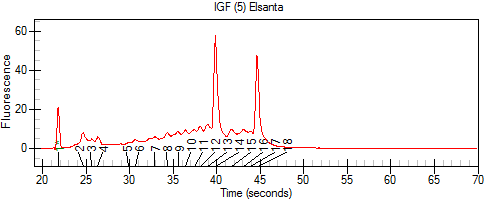

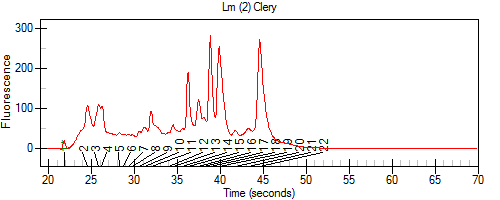


**Fig. S 10** **Electropherograms of Elsanta and Cléry DNase treated RNA samples (part 1** Ly = young leaf; Lm = mature leaf; P = petiole; R= root; sGF = small green fruit; lGF = large green fruit; WF = white fruit; RF = red fruit. The numbers between brackets indicate the biological replicate.
