# Supplementary material for: Identification and substrate prediction of new Fragaria x ananassa aquaporins and expression in different tissues and during strawberry fruit development
Source: Hortic Res. 2018 Apr 1;5:20. doi: 10.1038/s41438-018-0019-0 (PMC5880810; doi:10.1038/s41438-018-0019-0)
Supplement: Supplementary file 6 — Supplementary tables [file 41438_2018_19_MOESM6_ESM.docx]

**Article title**: Identification and substrate prediction of new *Fragaria x ananassa* aquaporins and expression in different tissues and during strawberry fruit development

**Journal**: Horticulture Research

**Authors**: Britt Merlaen, Ellen De Keyser and Marie-Christine Van Labeke

**Corresponding author**: Marie-Christine Van Labeke, Plant Production, Faculty of Bioscience Engineering, Ghent University, Coupure Links 653, 9000 Gent, Belgium Email: mariechristine.vanlabeke@ugent.be

**Supplementary tables** Table S1 – S7; References to this file

**Table S 1 Nanodrop data of Cléry DNase treated RNA samples.**

| Sample code  Cléry | Tissue | Biological replicate | Nucleic acid concentration (ng/µl) | A260/A230 | A260/A280 |
| --- | --- | --- | --- | --- | --- |
| Ly (1) | Young leaf | 1 | 977.97 | 2.31 | 1.97 |
| Ly (2) | Young leaf | 2 | 782.37 | 2.58 | 2.03 |
| Ly (3) | Young leaf | 3 | 778.44 | 2.50 | 2.02 |
| Lm (1) | Mature leaf | 1 | 2320.56 | 2.29 | 2.00 |
| Lm( 2) | Mature leaf | 2 | 1532.58 | 2.42 | 2.02 |
| Lm (3) | Mature leaf | 3 | 1220.00 | 2.32 | 2.03 |
| P (1) | Petiole | 1 | 602.94 | 2.82 | 2.05 |
| P (2) | Petiole | 2 | 686.46 | 2.62 | 2.04 |
| P (3) | Petiole | 3 | 723.62 | 2.71 | 2.05 |
| sGF (1) | Small green fruit | 1 | 462.76 | 2.68 | 1.96 |
| sGF (2) | Small green fruit | 2 | 577.86 | 2.69 | 2.05 |
| sGF (3) | Small green fruit | 3 | 1518.08 | 2.40 | 2.03 |
| lGF (1) | Large green fruit | 1 | 466.45 | 2.74 | 1.96 |
| lGF (2) | Large green fruit | 2 | 653.71 | 2.67 | 2.06 |
| lGF (3) | Large green fruit | 3 | 417.07 | 2.96 | 1.99 |
| WF (1) | White fruit | 1 | 552.04 | 2.82 | 2.04 |
| WF (2) | White fruit | 2 | 605.82 | 2.65 | 2.05 |
| WF (3) | White fruit | 3 | 979.68 | 2.51 | 2.04 |
| RF (1) | Red fruit | 1 | 274.54 | 3.02 | 2.02 |
| RF (2) | Red fruit | 2 | 552.18 | 2.69 | 2.03 |
| RF (3) | Red fruit | 3 | 693.39 | 2.62 | 2.04 |

**Table S 2 Nanodrop data of Elsanta DNase treated RNA samples.**

| Sample code Elsanta | Tissue | Biological replicate | Nucleic acid concentration (ng/µl) | A260/A230 | A260/A280 |
| --- | --- | --- | --- | --- | --- |
| Ly (1) | Young leaf | 1 | 450.46 | 2.92 | 1.98 |
| Ly (2) | Young leaf | 2 | 403.31 | 2.85 | 1.96 |
| Ly (3) | Young leaf | 3 | 1230.18 | 2.46 | 2.04 |
| Lm (1) | Mature leaf | 1 | 831.50 | 2.52 | 1.99 |
| Lm( 2) | Mature leaf | 2 | 526.06 | 2.67 | 2.01 |
| Lm (3) | Mature leaf | 3 | 1407.92 | 2.22 | 1.98 |
| P (1) | Petiole | 1 | 342.79 | 3.57 | 2.02 |
| P (2) | Petiole | 2 | 374.89 | 3.32 | 2.02 |
| P (3) | Petiole | 3 | 895.19 | 2.58 | 2.06 |
| sGF (4) | Small green fruit | 1 | 332.43 | 4.95 | 2.03 |
| sGF (5) | Small green fruit | 2 | 158.67 | -27.37 | 2.06 |
| sGF (6) | Small green fruit | 3 | 428.82 | 3.55 | 1.99 |
| lGF (4) | Large green fruit | 1 | 70.65 | -1.81 | 1.97 |
| lGF (5) | Large green fruit | 2 | 169.63 | 44.28 | 2.04 |
| lGF (6) | Large green fruit | 3 | 163.74 | 32.99 | 2.04 |
| WF (1) | White fruit | 1 | 364.28 | 3.16 | 2.00 |
| WF (2) | White fruit | 2 | 442.56 | 2.77 | 1.98 |
| WF (3) | White fruit | 3 | 468.32 | 2.75 | 1.97 |
| RF (1) | Red fruit | 1 | 508.83 | 2.78 | 2.02 |
| RF (2) | Red fruit | 2 | 411.68 | 3.00 | 1.99 |
| RF (3) | Red fruit | 3 | 864.27 | 2.51 | 2.05 |

**Table S 3 Primers, PCR conditions and origin tissue of the cDNA used for amplification of aquaporin fragments.** In case of fragment B, forward (F) and reverse primers (R) were developed on different sequences. Ta = annealing temperature

| Origin of primers | Forward primer (5’-3’) | Reverse primer (5’-3’) | Ta (°C) | Elongation time (s) | Tissue | Fragment |
| --- | --- | --- | --- | --- | --- | --- |
| FvPIP1;1 ^1^ | GTTCATCGCCACCTTTCTGT | GCCCTGATCACAACCACAT | 58 | 55 | Leaf | A (343 bp) |
| (F) Genbank Acc. No. DQ022749.1^2^  (R) FvPIP1;1 ^1^ | TGCTGAAATTATGGGCACCT | GCCCTGATCACAACCACAT | 58 | 38 | Leaf | B (563 bp) |
| FvPIP1;2 ^1^ | AGCTGGAATAGCTGAGTTTG | CCTAGTCTTGAATGGAATGG | 58 | 55 | Leaf | C (704 bp), D (701 bp) |
| FvPIP2;1 ^1^ | AGCTGTCATCGCAGAGTTCA | AGCAGGGTTGATACCAGTGC | 59 | 38 | Red fruit | E (575 bp) |

**Table S 4 Origin tissue of the cDNA used for RACE and 5’ and 3’ RACE primer sequences. Primer names after and to be used as described in Scotto-Lavino et al.**^3,4^

| Fragment | Tissue | 5' RACE GSP-RT (5’-3’) | 5' RACE GSP-1 (5’-3’) | 5' RACE GSP-2 (5’-3’) | 3' RACE GSP-1 (5’-3’) | 3' RACE GSP-2 (5’-3’) |
| --- | --- | --- | --- | --- | --- | --- |
| A | Leaf | GCTTCTTGTTGTAGATGATGT | CTGGCCAAGAAAAGGCCGAAA | GAAACAGAAAGGTGGCGATGA | TCGACTCAGTTGAGCTGACCAAA | GCCAGGAAGCTGTCCTTGACT |
| B | Leaf | ACCCAGACTTTGTGCTACTA | GGAGATACCGGCGGTGCAGTA | TAGCAATGAACTCTGCGATGA | GCTGAGATCATCGGCACCTTT | CGCCAGAGACTCCCATGTTCCT |
| C | Leaf | TTGATACCAGTTCCAGTGAT | GGCATCAGTGGCGGAGAAAA | TCCTTGCCAAGAAGAGACCA | TTGGCAACCATCCCCATCACT | TCGGACCATTCATTGGAGCTG |
| D | Leaf | CTTGCTAGGAACAGACCAA | TGTGTCCACCTGAGAACCAGCA | GACTCCCATCACAGTCAACACT | CCCATGCTTGGGATGACCAGT | TGCACTTGCTGCTTTCTACCACA |
| E | Red fruit | AAAGTCACAGCTGGGTTAAT | GTCGGCGTCGATCTGGGACT | AGGGTAGCAATGAACTCTGC | AAGAGATTCCCATGTTCCGGTTT | CGGCACTGGTATCAACCCTGCT |
| Genbank Acc. No. GW403182.1 | Red fruit | ACTACCCTTGGTGTAGCC | CACGGCAGCTCCGGCTATG | TCAAGGACAGCTTCCTGGCTA | AGCGCCAGAGACTCCCATGTT | AACCCAGCTAGGAGTCTTGGTG |

**Table S 5 Primers, cultivar and tissue used for amplification of each coding sequence and Genbank accession numbers for each coding sequence**

| Fragment | Forward primer (F) (5’-3’)  Reverse primer (R) (5’-3’) | Cultivar | Tissue | (Partial) Aquaporins | Genbank Acc. No. |
| --- | --- | --- | --- | --- | --- |
| Genbank Acc. No. GW403182.1 | (F) TCAAACGCAGTCGTTTTACC  (R) AAAGCTGTTGATTGTTGAAACC | Elsanta | Red fruit | FaPIP1;1-like (1-3) | KY453768 - KY453770 |
| Genbank Acc. No. GW403182.1 | (F) TCAAACGCAGTCGTTTTACC  (R) AAAGCTGTTGATTGTTGAAACC | Diamante | Leaf | FaPIP1;1-like (4-7) | KY453771 - KY453774 |
| C | (F) AAGCACCCAGAACCACAAAC  (R) AACTCTTCAATCCCAGTCACAA | Elsanta | Leaf | FaPIP1;2-1(-like(1)) & FaPIP1;2-2 | KY453775 - KY453776  KY453777 |
| D | (F) TGTGGCTACATTCTTGTTCCTC  (R) AATGGCCCTGATGACTACCA | Elsanta | Leaf | FaPIP1;3/partial &  FaPIP1;3-like/partial(1) | KY453778  KY453779 |
| D | (F) TGTGGCTACATTCTTGTTCCTC  (R) AATGGCCCTGATGACTACCA | Diamante | Leaf | FaPIP1;3-like/partial(2-4) | KY453780 - KY453782 |
| E | (F) TCTCTGAAACTCTGTGCTTTCTTG  (R) GTGGAAGCAACATCTTTCATTG | Elsanta | Leaf | FaPIP2;1-like(1) | KY453783 |
| E | (F) TCTCTGAAACTCTGTGCTTTCTTG  (R) GTGGAAGCAACATCTTTCATTG | Diamante | Leaf | FaPIP2;1-like(2) | KY453784 |
| E | (F) TCTCTGAAACTCTGTGCTTTCTTG  (R) GTGGAAGCAACATCTTTCATTG | Elsanta | Leaf | FaPIP2;1-1 | KY453785 |
| A | (F) CCAAAGCTCTATCCTCATCTTCTT  (R) TTGATTGTTGAAACGCTCACT | Diamante | Leaf | FaPIP2;1-1-like(1-4) | KY453786 - KY453789 |
| E | (F) TCTCTGAAACTCTGTGCTTTCTTG  (R) GTGGAAGCAACATCTTTCATTG | Diamante | Leaf | FaPIP2;1-1-like(5) | KY453790 |
| B | (F) TCCCAACTACATCCACTCACA  (R) CAAGTTTGGGTGGAAAATCC | Diamante | Leaf | FaPIP2;2 | KY453791 |
| B | (F) TCCCAACTACATCCACTCACA  (R) CAAGTTTGGGTGGAAAATCC | Elsanta | Leaf | FaPIP2;2-like/partial(1) & FaPIP2;2-like(2) | KY453792  KY453793 |

**Table S 6 Conserved residues in aquaporins. Ar/R stands for an aromatic amino acid and Arginine**

| Class | Location | Conserved residues | Posttranslational modification | Function | Reference(s) |
| --- | --- | --- | --- | --- | --- |
| All | Loop B | N P A (1) | - | Pore specificity | refs. 1 and 2 |
| All | Loop E | N P A (2) | - | Pore specificity | refs. 1 and 2 |
| All | N-terminus | A E F | - | Unknown | ref. 3 |
| PIP | - | Ar/R selectivity filter (F H T R) | - | Pore specificity | ref. 4 |
| PIP | Loop B | R/X K X S X X R/K | Phosphorylation of S | Opening of the water channel | refs. 5 and 6 |
| PIP | Loop D | S | Phosphorylation | Water channel activity regulation | ref. 6 |
| PIP | Loop D | H | Protonation | Closure of the water channel | ref. 7 |
| PIP | Loop D | L | - | Key blocking residue in closed state | ref. 8 |
| PIP2 | C-terminus | S^1^ X R/K S^2^ | Phosphorylation of S^1^ and S^2^ | S^1^: enhancement of the effect of phosphorylation of S in loop B  S^2^: trafficking to the plasma membrane | refs. 5, 6 and 9–11  ref. 12 |
| PIP2 | N-terminus | K | Methylation | Unknown | ref. 13 |
| PIP2 | N-terminus | D/E^1^ X D/E | Methylation of E^1^ | Trafficking between endoplasmic reticulum and plasma membrane | refs. 13 and 14 |
| TIP | - | Ar/R selectivity filter (a.o. H I A/G R) | - | Pore specificity | refs. 1 and 4 |
| TIP | Loop B | S/T | Phosphorylation | Opening of the water channel | ref. 5 |

**Table S 7 Overview of p-values resulting from Browne-Forsyth tests comparing the expression of the aquaporin groups between cultivars Cléry and Elsanta per tissue and per aquaporin-group (Table 7 )**. “–“ means that the test cannot be performed because at least one group has the sum of case weights less than or equal to 1. Values lower than the significance level of 0.05 are printed in bold. Ly = young leaf; Lm = mature leaf; P = petiole; sGF = small green fruit; lGF = large green fruit; WF = white fruit; RF = red fruit

| group | Ly | Lm | P | sGF | lGF | WF | RF |
| --- | --- | --- | --- | --- | --- | --- | --- |
| FaPIP1;1 | 0.290 | 0.501 | 0.376 | 0.060 | 0.259 | 0.137 | 0.411 |
| FaPIP1;2 | **0.049** | 0.193 | 0.094 | **0.011** | 0.093 | 0.317 | 0.265 |
| FaPIP1;3 | 0.181 | 0.677 | 0.116 | 0.706 | 0.416 | 0.119 | 0.471 |
| FaPIP2;1(a) | 0.770 | 0.095 | **0.030** | 0.619 | 0.057 | 0.218 | 0.806 |
| FaPIP2;1(b) | 0.134 | 0.150 | **0.039** | **0.008** | 0.121 | 0.447 | 0.119 |
| FaPIP2;2 | 0.066 | - | **0.012** | 0.128 | 0.303 | 0.793 | 0.736 |
| FaTIP(a) | 0.302 | 0.694 | 0.662 | 0.564 | 0.904 | 0.099 | 0.398 |
| FaTIP(b) | 0.257 | 0.977 | 0.262 | 0.069 | **0.025** | **0.036** | **0.007** |

## References

1 Hove RM, Bhave M. Plant aquaporins with non-aqua functions: Deciphering the signature sequences. Plant Mol Biol 2011; **75**: 413–430.

2 Sui H, Han BGG, Lee JKK, Walian P, Jap BKK. Structural basis of water-specific transport through the AQP1 water channel. Nature 2001; **414**: 872–878.

3 Zardoya R, Villalba S. A phylogenetic framework for the aquaporin family in eukaryotes. J Mol Evol 2001; **52**: 391–404.

4 Wallace IS, Roberts DM. Homology modeling of representative subfamilies of Arabidopsis major intrinsic proteins. Classification based on the aromatic/arginine selectivity filter. Plant Physiol 2004; **135**: 1059–1068.

5 Johansson I, Karlsson M, Johanson U, Larsson C, Kjellbom P. The role of aquaporins in cellular and whole plant water balance. Biochim Biophys Acta - Biomembr 2000; **1465**: 324–342.

6 Van Wilder V, Miecielica U, Degand H, Derua R, Waelkens E, Chaumont F. Maize plasma membrane aquaporins belonging to the PIP1 and PIP2 subgroups are in vivo phosphorylated. Plant Cell Physiol 2008; **49**: 1364–1377.

7 Tournaire-Roux C, Sutka M, Javot H et al. Cytosolic pH regulates root water transport during anoxic stress through gating of aquaporins. Nature 2003; **425**: 393–397.

8 Törnroth-Horsefield S, Wang Y, Hedfalk K et al. Structural mechanism of plant aquaporin gating. Nature 2006; **439**: 688–694.

9 Johansson I, Karlsson M, Shukla VK, Chrispeels M, Larsson C, Kjellbom P. Water Transport Activity of the Plasma Membrane Aquaporin PM28A Is Regulated by Phosphorylation. Plant Cell 1998; **10**: 451–459.

10 Johansson I, Larsson C, Ek B, Kjellbom P. The major integral proteins of spinach leaf plasma membranes are putative aquaporins and are phosphorylated in response to Ca2+ and apoplastic water potential. Plant Cell 1996; **8**: 1181–1191.

11 Törnroth-Horsefield S, Hedfalk K, Fischer G, Lindkvist-Petersson K, Neutze R. Structural insights into eukaryotic aquaporin regulation. FEBS Lett 2010; **584**: 2580–2588.

12 Prak S, Hem S, Boudet J et al. Multiple phosphorylations in the C-terminal tail of plant plasma membrane aquaporins: role in subcellular trafficking of AtPIP2;1 in response to salt stress. Mol Cell Proteomics 2008; **7**: 1019–1030.

13 Santoni V, Verdoucq L, Sommerer N, Vinh J, Pflieger D, Maurel C. Methylation of aquaporins in plant plasma membrane. Biochem J 2006; **400**: 189–197.

14 Zelazny E, Miecielica U, Borst JW, Hemminga MA, Chaumont F. An N-terminal diacidic motif is required for the trafficking of maize aquaporins ZmPIP2;4 and ZmPIP2;5 to the plasma membrane. Plant J 2009; **57**: 346–355.
